# Components of the Engulfment Machinery Have Distinct Roles in Corpse Processing

**DOI:** 10.1371/journal.pone.0158217

**Published:** 2016-06-27

**Authors:** Tracy L. Meehan, Tony F. Joudi, Allison K. Timmons, Jeffrey D. Taylor, Corey S. Habib, Jeanne S. Peterson, Shanan Emmanuel, Nathalie C. Franc, Kimberly McCall

**Affiliations:** 1 Department of Biology, Boston University, Boston, Massachusetts, United States of America; 2 The Scripps Research Institute, Department of Immunology and Microbial Science, La Jolla, California, United States of America; National Institute of Biological Sciences, Beijing, CHINA

## Abstract

Billions of cells die in our bodies on a daily basis and are engulfed by phagocytes. Engulfment, or phagocytosis, can be broken down into five basic steps: attraction of the phagocyte, recognition of the dying cell, internalization, phagosome maturation, and acidification. In this study, we focus on the last two steps, which can collectively be considered corpse processing, in which the engulfed material is degraded. We use the *Drosophila* ovarian follicle cells as a model for engulfment of apoptotic cells by epithelial cells. We show that engulfed material is processed using the canonical corpse processing pathway involving the small GTPases Rab5 and Rab7. The phagocytic receptor Draper is present on the phagocytic cup and on nascent, phosphatidylinositol 3-phosphate (PI(3)P)- and Rab7-positive phagosomes, whereas integrins are maintained on the cell surface during engulfment. Due to the difference in subcellular localization, we investigated the role of Draper, integrins, and downstream signaling components in corpse processing. We found that some proteins were required for internalization only, while others had defects in corpse processing as well. This suggests that several of the core engulfment proteins are required for distinct steps of engulfment. We also performed double mutant analysis and found that combined loss of *draper* and *αPS3* still resulted in a small number of engulfed vesicles. Therefore, we investigated another known engulfment receptor, Crq. We found that loss of all three receptors did not inhibit engulfment any further, suggesting that Crq does not play a role in engulfment by the follicle cells. A more complete understanding of how the engulfment and corpse processing machinery interact may enable better understanding and treatment of diseases associated with defects in engulfment by epithelial cells.

## Introduction

Engulfment by epithelial cells is essential for the health and maintenance of several organs including the retina, lungs, and kidney [1–4]. Improper clearance can result in or exacerbate serious conditions such as retinitis pigmentosa, age-related macular degeneration, and asthma [3–5]. Despite the importance of epithelial cells in engulfment, the molecular changes within these cells that occur during engulfment are only now beginning to be elucidated. Much of the recent progress has identified the core proteins that are required for engulfment in different cell types and across species.

Several proteins required for engulfment have been identified and investigated in *C*. *elegans*, *Drosophila*, and mammals. Two well-known engulfment receptors are Draper/Ced-1 and integrins. In *C*. *elegans*, these receptors act in two partially parallel pathways: Ced-1 working with Ced-6 and Ced-7 and integrins working upstream of Ced-2/5/12 [6, 7], both of which can activate the small GTPase Rac1 [8]. In *Drosophila*, Rac1 is activated by Ced-5/Ced-12, but it is not known what acts upstream of Ced-5/Ced-12 [9, 10]. In *Drosophila* hemocytes, integrins have recently been shown to be required for engulfment [11–14] and Draper has been shown to work in parallel to integrins in these cells [11]. Ced-12 has been shown to activate Rac1 in conjunction with another GEF, DRK/DOS/SOS, both acting downstream of Draper in phagocytic glia [10]. Conversely, in *Drosophila* hemocytes, Draper and Ced-12 were found to act in parallel pathways [15], suggesting that the engulfment pathways may differ between cell types. In mammals, the activation of Rac1 by the Ced-12 ortholog Dock180/ELMO1 has been studied extensively, although the Dock180/ELMO1 complex is usually activated by another engulfment receptor, Bai1 [16, 17]. Although Bai1 orthologs have not been reported in *Drosophila*, other conserved engulfment receptors include the CD36 family members Crq and Debris buster [18–21]. Determining the specific roles of these proteins during engulfment is an active area of investigation.

Once the engulfed material has been internalized, it is degraded through the corpse processing pathway. The corpse processing pathway has been well-characterized in *C*. *elegans* and mammals, indicating that the machinery is conserved across species. In *C*. *elegans*, phagocytic cup formation is dependent on the receptor Ced-1 and large GTPase Dynamin [22, 23]. The nascent phagosomes fuse to early and late endosomes for phagosome maturation, using the small GTPases Rab5 and Rab7, respectively [23, 24]. The late endosomes then fuse to lysosomes, mixing their contents together and degrading the engulfed material [25–28]. These events are conserved in mammals, although there are a large number of receptors in addition to Ced-1 homologues. In this study, we refer to “engulfment” as the complete process, from recognition through acidification. We refer to “corpse processing” as the process including only phagosome maturation and acidification, after internalization and formation of the phagosome. Lastly, we refer to “phagosome maturation” as the process encompassing vesicle association with Rab5- and/or Rab7-GFP.

The studies on phagosome maturation have largely been centered around the function of Rab GTPases. However, recent work has also uncovered a role for specific lipid composition changes on the phagosome surface. Phosphatidylinositol 3-phosphate (PI(3)P) serves as a signaling molecule for several different cellular processes including phagosome maturation in mammals and *C*. *elegans* [29, 30]. Interestingly, in *C*. *elegans*, PI has been shown to be phosphorylated and de-phosphorylated in cyclic waves for proper phagosome maturation, requiring two kinases and one phosphatase [29]. Phosphatidylinositol 4,5-bisphosphate (PI(4,5)P_2_) associates with the unsealed phagocytic cup while PI(3)P associates with newly sealed phagosomes [31]. Lipid composition, specifically the presence of PI(3)P or PI(4,5)P_2_, can serve as a localization cue for proteins required for phagosome maturation [31]. In *Drosophila*, phagosome maturation has been characterized in hemocytes processing engulfed bacteria [32] and epidermal cells processing degraded dendrites [20]. We have recently demonstrated that phagosome maturation markers can be detected during engulfment of dead cells by epithelial follicle cells in the *Drosophila* ovary [33]. Throughout this study, we refer to PI(3)P, Rab5, and Rab7 as markers of the canonical corpse processing pathway.

While much is known about the canonical corpse processing machinery, how the core engulfment machinery (phagocytic receptors and downstream proteins) interacts with the corpse processing machinery is not as well understood. The phagocytic receptor Draper/Ced-1 has been shown to play different roles depending on the cell type. In *C*. *elegans* and some cell types in *Drosophila*, Draper/Ced-1 is required for internalization and corpse processing [34–37]. However, in hemocytes and epidermal cells of *Drosophila*, Draper is required only for corpse processing [20, 38]. Another phagocytic receptor, Croquemort, is required for internalization in hemocytes [18] and corpse processing in epidermal cells of *Drosophila* [20]. This indicates that the engulfment machinery does interface with the corpse processing machinery, and that these interactions can differ by cell type.

Here, we investigate how the engulfment machinery interacts with the corpse processing machinery in the epithelial follicle cells of the *Drosophila* ovary. The *Drosophila* ovary serves as an excellent model for studying cell death and engulfment by epithelial cells. The *Drosophila* ovary is made of chains of progressively developing egg chambers. Each mid-stage egg chamber consists of the germline-derived nurse cells and oocyte and surrounding epithelial follicle cells. Apoptotic cell death in mid-oogenesis can be induced easily by starvation [39–45]. When flies are deprived of nutrients, the germline-derived nurse cells undergo apoptosis and the surrounding somatically-derived follicle cells enlarge and engulf the dying material [39, 45]. We have uncovered some of the molecular changes required for follicle cells to engulf the dying germline [14, 45]. We found that the phagocytic receptor Draper activates the JNK pathway, working in a positive feedback loop [45]. Recently, we have found that the integrin heterodimer, αPS3/βPS, is also required for engulfment but not JNK activation [14].

In this study, we show that the epithelial follicle cells utilize the canonical corpse processing pathway to degrade the dying germline. We find that Draper is present on nascent phagosomes whereas integrins are not. Moreover, Draper functions in both internalization and corpse processing in the follicle cells, whereas integrins are required only for internalization and activation of downstream signaling molecules. Surprisingly, we found three distinct categories of mutant phenotypes: those with defects in internalization only, those with defects in internalization and phagosome maturation, and those with defects in internalization, phagosome maturation, and acidification. We also found that combined loss of two phagocytic receptors, *draper* and *αPS3*, still resulted in a small number of engulfed vesicles, which was not further affected by the loss of another phagocytic receptor, Crq. However, we found that Crq may be required to promote nurse cell death. This suggests that Draper and integrins are the major phagocytic receptors on the follicle cells. Our findings also suggest that several engulfment genes may have dual roles for internalization and corpse processing, while others are only required for internalization. This work also indicates a possible explanation for why an engulfing cell utilizes multiple engulfment receptors. Each receptor may have specific, non-overlapping functions that are crucial for successful engulfment.

## Materials and Methods

### Fly strains and manipulations

All strains were reared on standard cornmeal molasses fly food at 25°C unless otherwise indicated. For starvation experiments, adult flies were placed in new vials containing fly food supplemented with freshly made yeast paste for 1.5–2 days and transferred to apple juice agar vials overnight [14]. All strains were obtained from Harvard TRiP [46], the Bloomington Stock Center or Vienna *Drosophila* Resource Center unless otherwise indicated (Table 1). *UAS-Rab5GFP* and *UAS-Rab7GFP* were recombined with the FC specific driver *GR1-GAL4* (Trudi Schϋpbach, Princeton University, NJ, USA; [33, 45, 47] and were crossed to dsRNA lines for corpse processing analysis. Some dsRNA lines were lethal, so *GR1-GAL4* was combined with *tubulin-GAL80*^*ts*^ and flies were reared at 18°C. Progeny with *tubulin-GAL80*^*ts*^ were transferred to 29°C for 2 days to inactivate GAL80. Crosses using *αPS3* dsRNA were also transferred to 29°C for 2 days as described previously [14]. All other lines were reared, conditioned, and starved at 25°C. *drpr*^*Δ5*^ [14, 45, 48] was provided by Estee Kurant (Technion-Israel Institute of Technology, Haifa, Israel), and *crq*^*KO*^ is described in [20]. The *UAS-mCherry*::*2XFYVE* line [49] was provided by Amy Kiger (University of California, San Diego, CA, USA).

**Table 1:** Candidate dsRNA Screen Results.

Bloomington or Vienna #	Allele	Gene	Germline engulfment defects	Other defects
28556	HM05042	*Ced-12*^*#*^	***	**—**
3799	GD1529	*shi*^*#*^	***	Excessive FC growth at terminal end of egg chamber
105971	KK101444	*shi*	***	n.d.
34832	HMS00147	*Rab5*^*#*^	***	**—**
33734	GD10124	*dor*^*#*^	***	**—**
27519	JF02669	*Rab8*	***	n.d.
34373	HMS01363	*Rab8*	***	n.d
28021	JF02855	*Cdc42*	***	Double layer at the posterior
27722	JF02804	*kayak*^*#*^	**	**—**
100708	KK108017	*Src42A*^*#*^	**	**—**
27051	JF02377	*Rab7*^*#*^	*	**—**
33617	HMS00010	*Fak56D*	*	**—**
28342	JF02978	*Rab35*	**—**	**—**
28701	JF03117	*Rab2*	**—**	**—**
34922	HMS01271	*Rab2*	**—**	**—**
31688	JF01861	*Rab9*	**—**	**—**
42942	HMS02635	*Rab9*	**—**	**—**
28708	JF03135	*Rab14*	**—**	**—**
34654	HMS01130	*Rab14*	**—**	**—**
28513	JF03133	*shi*	**—**	**—**
36921	HMS00154	*shi*	**—**	**—**

Genes disrupted, Bloomington and Vienna (v) stock numbers, and any associated TRiP numbers are listed. The phenotypes are based on a 3-star system, where 3 stars (***) is the most severe, 2 stars (***) is moderate, and 1 star (*) is the least severe, and is usually associated with weak and/or variable lines. Two dashes (—) indicate no phenotype, n.d. is not determined. A pound sign (#) indicates the lines that are analyzed in this paper.

### Antibody staining and microscopy

For antibody staining, flies were dissected in Grace’s medium and ovaries were fixed and stained as described previously [44]. For LysoTracker staining, flies were dissected in PBS, incubated in 1:600 LysoTracker for 5 minutes, rinsed and washed in PBS, and finally fixed in PBS, heptane, and paraformaldehyde for 20 minutes. At this point, samples were treated as with standard antibody staining. All samples were mounted in VectaShield with DAPI (Vector Labs). Primary antibodies used were: cleaved α-Dcp-1 (1:100, Cell Signaling), α-Dlg (1:100, Developmental Studies Hybridoma Bank (DSHB)), α-Draper (1:50, DSHB), α-αPS3 (1:1000, [14]), and α-β-Gal (1:400, Promega). Secondary antibodies used were goat-α-rabbit Cy3 and goat-α-mouse Alexa Fluor 647 (Jackson ImmunoResearch), each at 1:100. Egg chambers were imaged on an Olympus FV10i confocal microscope, images were processed using ImageJ and Adobe Photoshop, and figures were made using Adobe Illustrator and Graphpad Prism.

### Engulfment quantification

We quantified vesicle uptake by Dcp-1 staining as previously described [14]. A central slice was counted for each egg chamber. At least three egg chambers were analyzed for each phase (phases 1–4) and genotype, unless otherwise indicated. The number of Rab7GFP-positive or LysoTracker-positive vesicles were counted in the same way. We quantified Rab7GFP-positive vesicles using the Dcp-1/Rab7GFP merge, to verify that the vesicles quantified contained engulfed germline. To determine if phagosome maturation was occurring properly, the ratios were then calculated by dividing the average number of Rab7-positive by the average number of Dcp-1-positive vesicles. *P* values were determined using two-tailed *t* tests.

## Results

### Vesicles engulfed by the follicle cells are processed using the canonical corpse processing pathway

**Fig 1:**
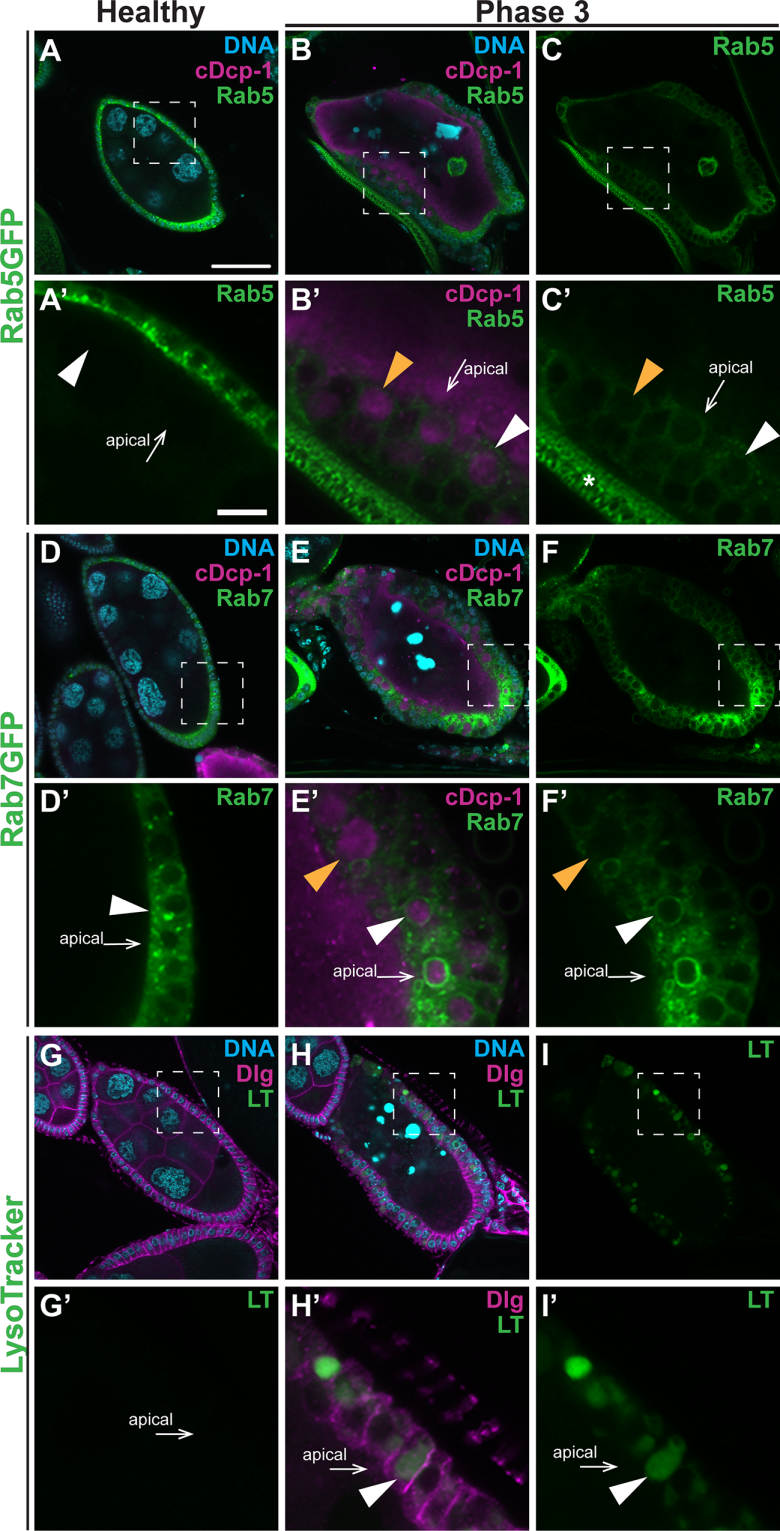
The active Dcp-1 antibody marks engulfed vesicles that are processed in an endocytic fashion. Mid-stage egg chambers stained with DAPI (cyan) and antibodies against cleaved Dcp-1 (magenta). (A-C’) Egg chambers expressing Rab5GFP (*UAS-GAL4/+; GR1-GAL4*, *UAS-Rab5GFP/UAS-luciferase*^*dsRNA*^) show normal engulfment and Rab5 association. (A-A’) In healthy egg chambers, Dcp-1 is not detectable and small Rab5GFP-positive vesicles are enriched at the apical region of the follicle cells (Rab5 only in zoom in A’). (B-C’) In dying phase 3 egg chambers, the germline is Dcp-1-positive. Many of the engulfed vesicles are also Dcp-1-positive (zoom in B’). (C, zoom in C’) Some vesicles in a phase 3 egg chambers become Rab5-positive (white arrowhead). The association does not make a complete circle around the vesicle, but rather several puncta associate with the vesicle. Several Rab5-negative vesicles are also still present (orange arrowhead). * indicates autofluorescence of dorsal appendage. (D-F’) Egg chambers expressing Rab7GFP (*UAS-GAL4/+; GR1-GAL4*, *UAS-Rab7GFP/UAS-luciferase*^*dsRNA*^) show normal engulfment and Rab7 association. (D-D’) In healthy egg chambers, Dcp-1 is not detectable and small Rab7GFP-positive vesicles are enriched at the apical region of the follicle cells (Rab7 only in zoom in D’). (E-F’) In phase 3 egg chambers, the germline is Dcp-1-positive. Many of the engulfed vesicles are also Dcp-1-positive. (E, zoom in E’) Some vesicles in phase 3 egg chambers become Rab7-positive (F, zoom in F’, white arrowhead). The association is seen as a bright circle completely surrounding a Dcp-1-positive vesicle (E’). Several Rab7-negative vesicles are also present (orange arrowhead). (G-I’) Egg chambers stained with LysoTracker (genotype: *UAS-GAL4/+; GR1-GAL4*, *UAS-Rab5GFP/UAS-luciferase*^*dsRNA*^) (G-G’) In healthy egg chambers, LysoTracker is not detectable within the germline or surrounding follicle cells (LysoTracker only in zoom in G’). (H-I’) In phase 3 egg chambers, the germline is not LysoTracker-positive but many of the engulfed vesicles are LysoTracker-positive (H, I, zooms in H’, I’, vesicle indicated by white arrowhead). Egg chamber scale bar is 50μm. Zoom scale bar is 10μm.

When flies are deprived of protein, the germline cells in some egg chambers in mid-oogenesis undergo apoptosis and are subsequently engulfed by the surrounding epithelial follicle cells [14, 39, 45, 50]. We previously analyzed dying egg chambers based on the state of nurse cell chromatin and characterized engulfment into five distinct phases [14, 45]. Throughout this paper, we show phase 0 (or healthy), phase 3 (mid-dying), and phase 5 (terminal) egg chambers. The nurse cell chromatin in phase 0 (healthy) egg chambers is dispersed (Fig 1A, 1D and 1G), whereas the nurse cell chromatin in phase 3 egg chambers is highly condensed (Fig 1B, 1E and 1H). By phase 5, little to no lingering nurse cell nuclear fragments remain.

To visualize phagosome maturation, we used existing transgenic lines expressing GFP fusions to Rab5 [51] and Rab7 [52] in conjunction with an antibody raised against cleaved caspase Dcp-1. We have found that this antibody against active Dcp-1 marks the dying germline and the subsequent engulfed material [14, 33, 50]. Studies in *C*. *elegans* and *Drosophila* have shown that early phagosomes are labeled with Rab5 whereas late phagosomes are labeled with Rab7 [20, 23]. We found that both Rab5 and Rab7-labeled endosomes accumulated near the apical surface of the follicle cells in healthy and early dying egg chambers (Fig 1A–1A’, 1D–1D’). In dying egg chambers, Rab5 and Rab7 were detected surrounding several of the engulfed Dcp-1-positive vesicles. As Rab5 and Rab7 are known to label maturing phagosomes, this indicates maturation of the Dcp-1-positive phagosomes. Rab5GFP was seen as puncta around Dcp-1-positive vesicles (Fig 1B–1C’), whereas Rab7GFP completely surrounded the Dcp-1-positive vesicles (Fig 1E–1F’). The last step of corpse processing is fusion with lysosomes, which can be visualized using LysoTracker, an indicator for compartments of high acidification. We found that there were no large LysoTracker-positive vesicles in healthy egg chambers (Fig 1G–1G’), but there were several LysoTracker-positive vesicles seen by phase 3 (Fig 1H–1I’). Our results are consistent with the previous studies performed in *C*. *elegans* and *Drosophila*, indicating that the dying germline is internalized and processed using the canonical corpse processing pathway.

### Engulfment receptors have distinct expression patterns during corpse processing

**Fig 2:**
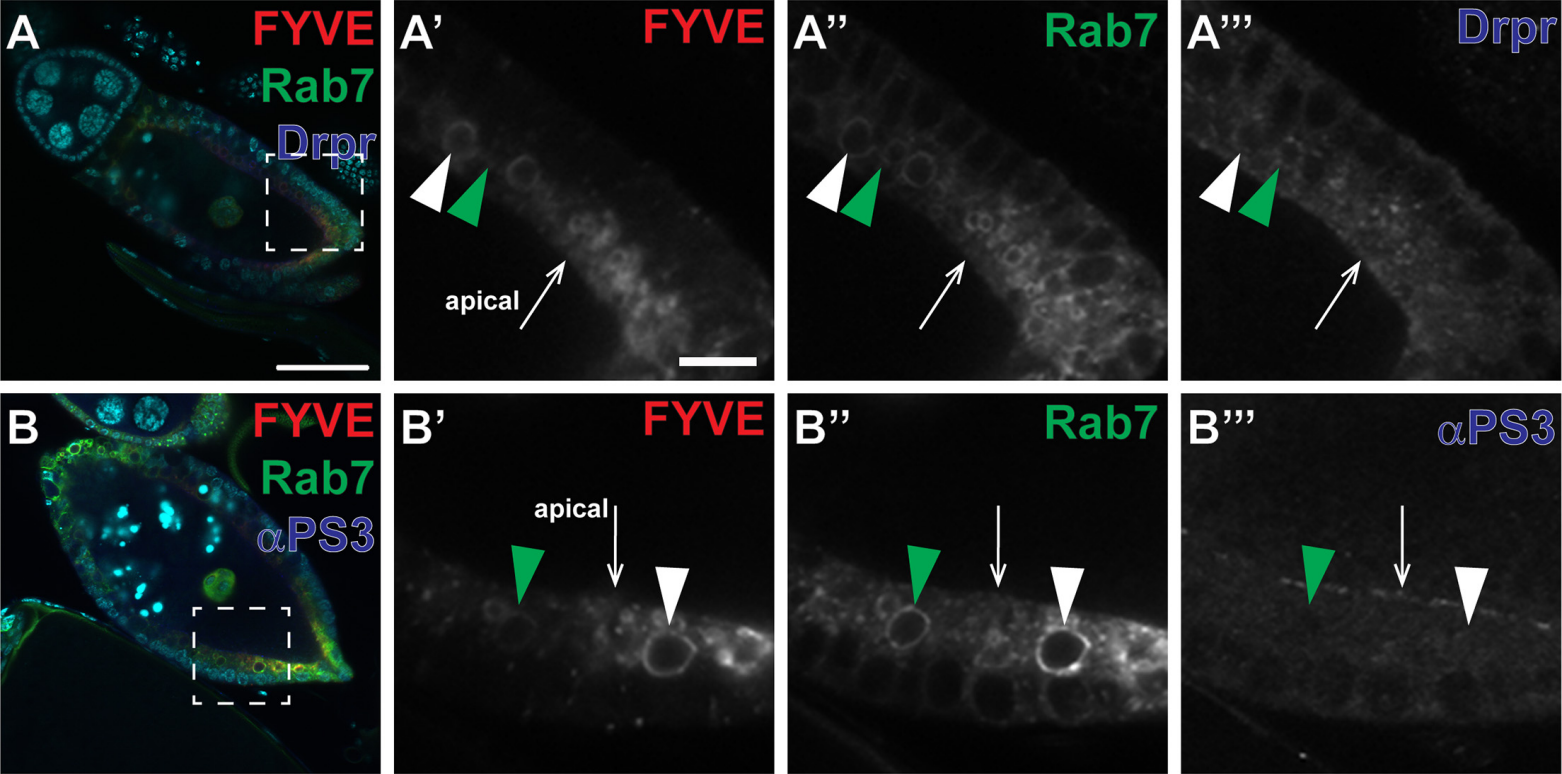
Maturing phagosomes are marked with FYVE and Draper, but not integrins. Dying mid-stage egg chambers expressing FYVE (red) and Rab7GFP (green) labeled with DAPI (cyan) and antibodies (blue) against Draper (A) or αPS3 (B). (A-A”‘) A mid-stage dying egg chamber (*UAS-GAL4/FYVE-mCherry; GR1-GAL4*, *UAS-Rab7GFP/+*) stained with α-Draper shows normal engulfment, Rab7 association, and Draper enrichment. (A’-A”‘) Single channel zooms show that FYVE-mCherry co-localizes with some Rab7GFP-positive vesicles (white arrowhead) and clusters near the apical surface of the follicle cells. Several of the FYVE- and Rab7-positive vesicles are also positive for Draper (white arrowhead). Some FYVE- and Rab7-negative vesicles were positive for Draper (green arrowhead). (B-B”‘) A mid-stage dying egg chamber (*UAS-GAL4/FYVE-mCherry; GR1-GAL4*, *UAS-Rab7GFP/+*) stained with anti-αPS3 shows normal engulfment, Rab7 association, and αPS3 enrichment. (B’-B”‘) Single channel zooms show that αPS3 is present only on the apical surface and not within the cell. A Rab7-positive vesicle with low FYVE (green arrowhead) and a Rab7- and FYVE-positive vesicle (white arrowhead) are shown, both negative for αPS3. Egg chamber scale bar is 50μm. Zoom scale bar is 10μm.

Previously, we showed that Draper is enriched, internalized, and required within engulfing follicle cells [45]. Recently, we also found that loss of Draper resulted in a larger number of engulfed vesicles by phase 4 of engulfment than loss of integrins [14]. The *C*. *elegans* ortholog of Draper, CED-1, has previously been shown to be present not only on the phagocytic cup, but also on phagosomes within the engulfing cell [53], indicating that CED-1 becomes internalized with the engulfed material. The increase in internalized vesicles in the *Drosophila* ovary in *draper* mutants could be due to an increase in internalization by a Draper-independent mechanism, or because vesicles accumulate over time because of a defect in corpse processing. To distinguish between these two possibilities, we first investigated the expression patterns of Draper and integrins with respect to the phagosome maturation markers phosphatidylinositol 3-phosphate (PI(3)P) and Rab7. We obtained transgenic lines expressing mCherry fused to FYVE [49], which targets mCherry to membranes enriched with PI(3)P. PI(3)P has been shown to mark vesicles immediately after the phagosome is sealed [31], although it is not clear when PI(3)P is removed from the phagosome. We found that not all Rab7-positive vesicles were positive for PI(3)P (Fig 2A’–2A”, white vs. green arrowheads). Several vesicles were positive for PI(3)P, Rab7, and Draper (Fig 2A’–2A”‘). Many of the vesicles that were positive for PI(3)P were also positive for Draper, suggesting there may be a relationship between lipid changes and Draper presence. Some vesicles, however, were Draper-positive and were negative for PI(3)P and Rab7 (Fig 2A–2A”, green arrowhead). These vesicles may be less mature, and would later become PI(3)P and Rab7-positive, or may be endosomes recycling Draper to lysosomes or back to the surface. Conversely, αPS3 was only present on the apical surface and did not localize to PI(3)P- or Rab7-positive vesicles (Fig 2B’–2B”‘). These data are consistent with previous studies, as we find that PI(3)P co-localized with vesicles near the apical surface, suggesting that they have begun to mature. Our data also provide further information regarding when PI(3)P is removed from the phagosome. We show here that PI(3)P is present on several Rab7-positive vesicles, but not all of them, indicating that PI(3)P may be removed before acidification. These results also show that Draper is present on the phagocytic cup and the newly internalized phagosomes, whereas integrins remain on the apical surface. This suggests that Draper becomes internalized, as seen previously [45], and may be required for corpse processing as in other systems [37, 38]. Conversely, integrins are not internalized, which may indicate differences in how Draper and integrins function within an engulfing cell. These two possibilities are investigated further below.

**Fig 3:**
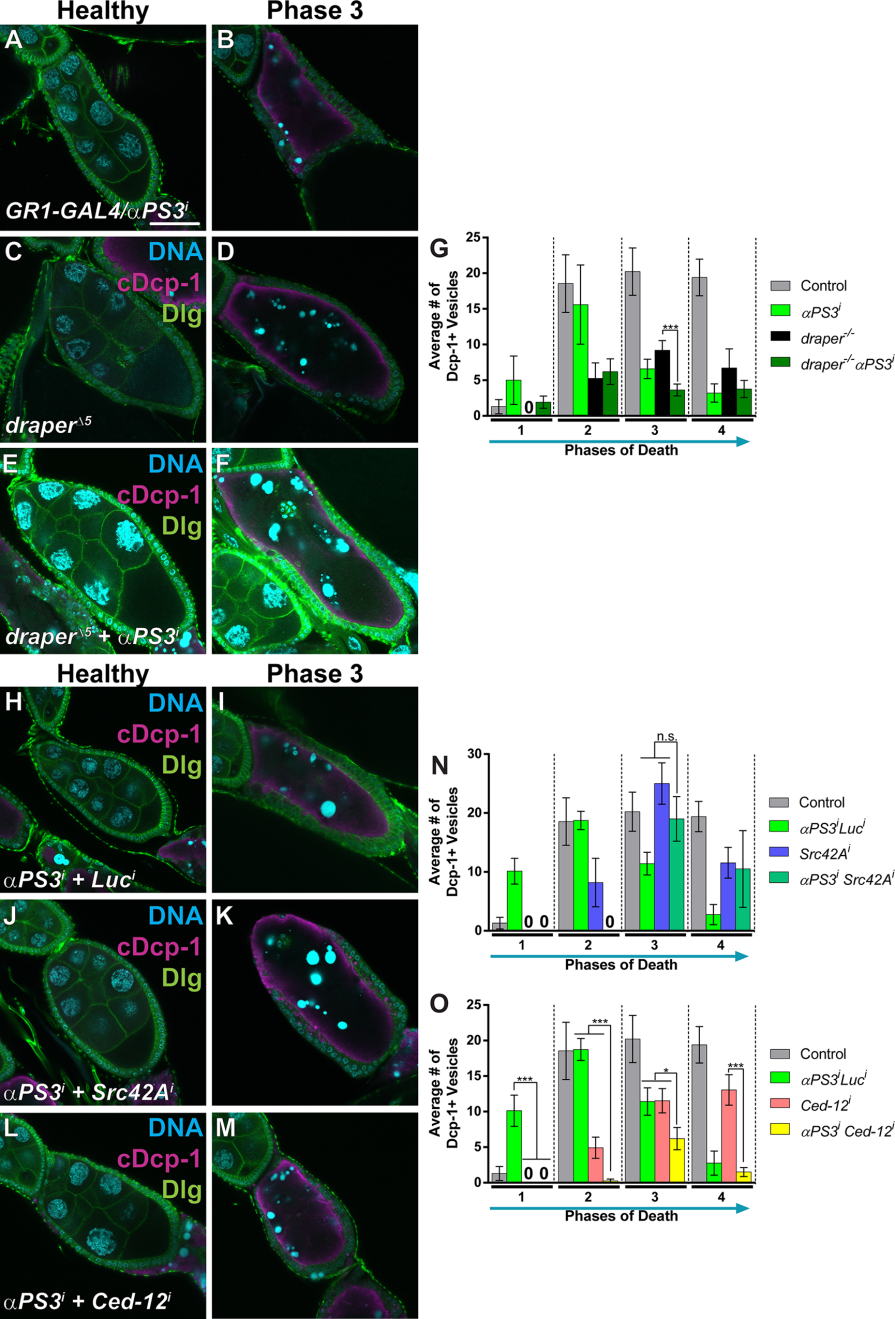
*αPS3* engulfment defects are enhanced by *draper* and *Ced-12*, but not by *Src42A*. Healthy and dying mid-stage egg chambers from the indicated genotypes stained with DAPI (cyan) and antibodies against cleaved Dcp-1 (magenta) and Discs large (Dlg, green). (A-B) *αPS3* RNAi (*GR1-GAL4/UAS-αPS3*^*dsRNA*^) and (C-D) *draper*^*Δ5*^ egg chambers show very little follicle cell growth and vesicle uptake as previously shown [14, 45]. (E-G) Combined loss of *draper* and αPS3 (*draper*^*Δ5*^ *UAS-αPS3*^*dsRNA*^*/draper*^*Δ5*^, *GR1-GAL4*) shows stronger defects than either single mutant, but only in phase 3. (H-I) *αPS3* RNAi + *luciferase* RNAi (*UAS-GAL4/+; GR1-GAL4*, *UAS-αPS3*^*dsRNA*^*/UAS-luciferase*^*dsRNA*^), shows slightly weaker defects in engulfment when compared to loss of αPS3 alone (shown in A-B and [14]). (J-K) Combined loss of *αPS3* and *Src42A* (*UAS-GAL4/ UAS-Src42A*^*dsRNA*^*; GR1-GAL4*, *UAS-αPS3*^*dsRNA*^*/+*) shows no additional defects compared to loss of *αPS3* alone shown in H-I. (L-M) Combined loss of *αPS3* and *Ced-12* (*UAS-GAL4/+; GR1-GAL4*, *UAS-αPS3*^*dsRNA*^*/UAS-Ced-12*^*dsRNA*^) shows some engulfment in phase 3 egg chambers, but no engulfment in earlier phases. (G, N-O) Quantification of the average number of Dcp-1-positive vesicles for each phase of death per central slice, for the indicated genotypes. All data are mean ± s.e.m. At least three egg chambers were quantified for each genotype and phase in G, N and O, except those noted here. For G, N and O, 47 egg chambers were quantified for the control (*UAS-GAL4/+; GR1-GAL4*, *UAS-Rab5GFP/UAS-luciferase*^*dsRNA*^ and *UAS-GAL4/+; GR1-GAL4*, *UAS-Rab7GFP/UAS-luciferase*^*dsRNA*^). For G, 44 egg chambers were quantified for *αPS3*^*dsRNA*^; 46 for *draper*^*Δ5*^; 85 for *draper*^*Δ5*^ *αPS3*^*dsRNA*^. For N, 78 egg chambers were quantified for *αPS3*^*dsRNA*^ *luciferase*^*dsRNA*^; 32 for *Src42A*^*dsRNA*^; and 6 for *αPS3*^*dsRNA*^ *Src42A*^*dsRNA*^. For O, 78 egg chambers were quantified for *αPS3*^*dsRNA*^ *luciferase*^*dsRNA*^; 95 for *Ced-12*^*dsRNA*^; and 22 for *αPS3*^*dsRNA*^ *Ced-12*^*dsRNA*^. Phase 1 for *Src42A* alone, and *Src42A* + *αPS3*, and phase 2 for *Src42A* + *αPS3* have less than 3 egg chambers quantified. Two-tailed *t*-tests were performed: ***–*P*<0.005, **–*P*<0.01, *–*P*<0.05.

To further investigate the engulfment pathways, we used double mutant analysis. First we generated double mutants with *draper*^*Δ5*^ and follicle cell-specific RNAi against *αPS3*. Engulfment was visualized and quantified (Fig 3A–3G) using the cleaved Dcp-1 antibody to mark engulfed vesicles, and anti-Discs Large to monitor the growth of follicle cell membranes. Loss of *draper* and *αPS3* simultaneously resulted in stronger engulfment defects in phase 3 compared to single mutants (Fig 3A–3G). Loss of *draper* normally results in some accumulation of vesicles beginning in phase 3, and the double mutant phenotypes suggest this could partially result from *draper*-independent engulfment mediated by the integrin pathway.

We also wanted to determine whether the engulfment genes *Ced-12* and *Src42A* functioned in the same pathway as *αPS3*. To analyze these genes in combination with *αPS3* by RNAi, it was necessary to recombine *UAS-αPS3-dsRNA* and *GR1-GAL4* onto the same chromosome. While this recombinant did have engulfment defects, we found that it showed a weaker phenotype in the earlier phases of engulfment (Fig 3H and 3I) compared to the trans-heterozygote (*UAS-αPS3-dsRNA*/*GR1-GAL4*, Fig 3A and 3B, [14]). Previously, we showed that αPS3 is required for engulfment starting in phase 2 [14], however the recombinant did not show engulfment defects until phase 3 (Fig 3N and 3O). To determine if the *GR1-GAL4*, *αPS3 dsRNA* recombinant was effectively knocking down *αPS3*, we used antibody staining to compare αPS3 levels between the recombinant and the trans-heterozygote. Both showed reduced αPS3 staining compared to wild-type (S1 Fig). These results suggest that there may be compensation by another pathway when αPS3 is continuously knocked down when maintaining a permanent stock, or the recombinant may have a less efficient knockdown of αPS3.

Double mutants were generated by crossing the *GR1-GAL4*, *UAS-αPS3-dsRNA* recombinant to either a control dsRNA line (*luciferase*, Fig 3H and 3I) or dsRNA lines against genes of interest (*Ced-12* and *Src42A*). Combined loss of *Src42A* and *αPS3* resulted in poor viability, but the egg chambers did not have added defects compared to single knockdowns (Fig 3G, 3H and 3L), suggesting that much like *C*. *elegans*, integrins may act through Src42A for engulfment. Combined loss of *Ced-12* and *αPS3* resulted in considerably fewer vesicles taken up during phases 1 and 2 compared to single knockdowns, although there was only a moderate difference in phase 3 and no difference in phase 4 (Fig 3L, 3M and 3O). This suggests that Ced-12 may be activated in an integrin-independent manner initially, but integrins act through Ced-12 later in engulfment, unlike the pathway described in *C*. *elegans* in which integrins work through Ced-12 from the initiation of engulfment. One possibility is that Ced-12 is initially activated by another engulfment protein, perhaps Draper, which has been shown to activate Ced-12 previously (Lu, 2014), and acts in early phases of engulfment, as our data suggest. Together, these results suggest that the role of integrins may be to bind to the apoptotic cell and activate downstream signaling (via Src42A and Ced-12) while Draper may be required for internalization and processing as in other systems [37, 38].

**Fig 4:**
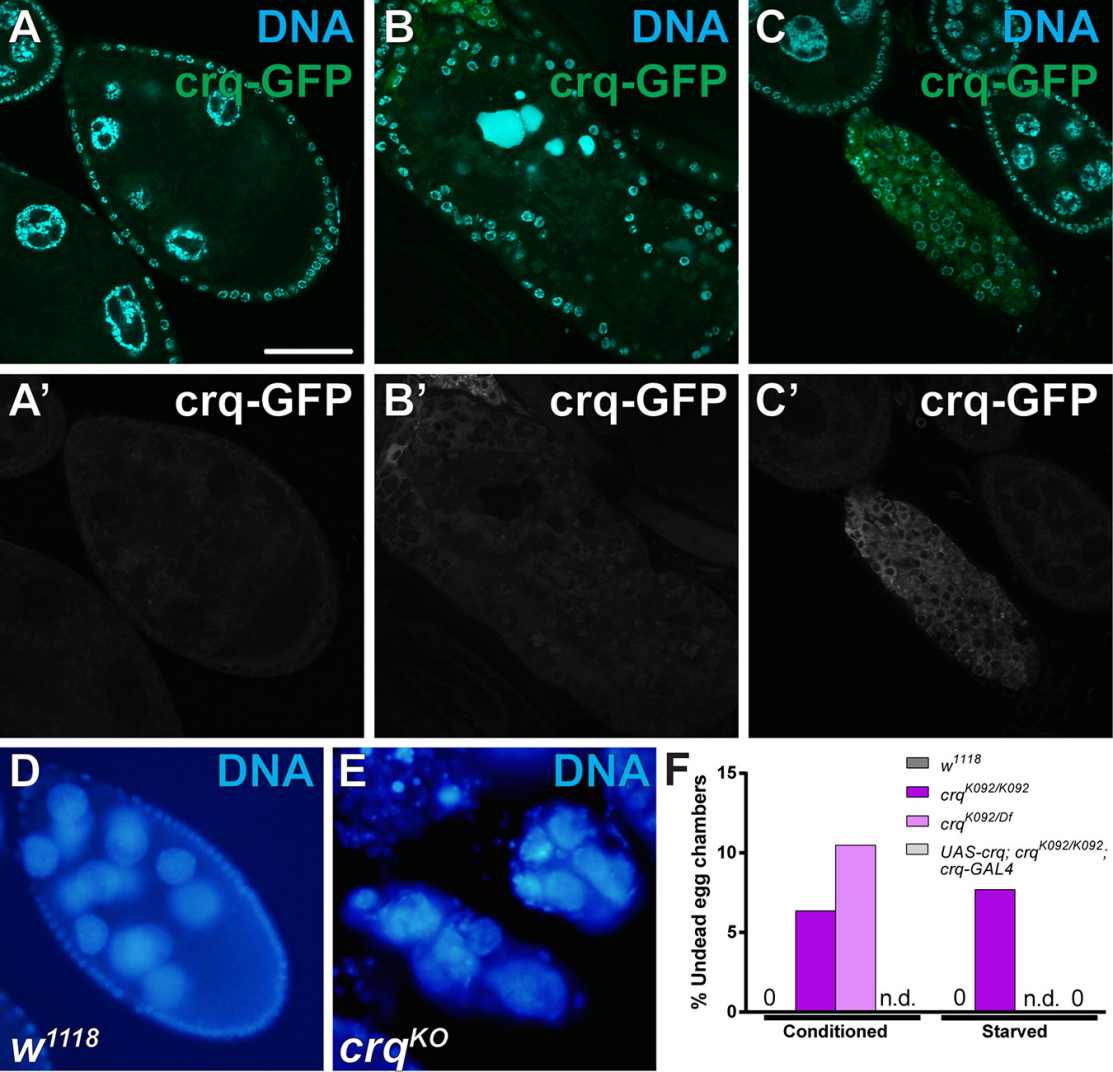
Croquemort becomes enriched in the follicle cells during engulfment and has defects in follicle cell health. (A-C) Egg chambers expressing *UAS-GFP* driven by *Crq-GAL4* (*Crq-GAL4/CyO; UAS-mCD8-GFP/TM2*) stained with DAPI (cyan). (A-A’) Healthy egg chambers show little to no Crq expression in the germline or follicle cells. (B-B’) Phase 3 egg chambers show an increase in Crq expression in the follicle cells. (C-C’) Phase 5 egg chambers show considerably more Crq expression in the remaining follicle cells. (D-E) Egg chambers from starved flies stained with DAPI (cyan). (D) A wild-type egg chamber (*w*^*1118*^) has a germline surrounded by a monolayer of healthy follicle cells. (E) *crq* null egg chambers (*crq*^*KO*^) show undead germline with no remaining healthy follicle cells. (F) Quantification of the number of “undead” egg chambers per 100 ovarioles in the indicated genotypes. Scale bar is 50μm.

Despite the importance of Draper and αPS3 during engulfment [11, 12, 14, 45, 48, 54, 55], loss of both receptors did not result in a complete block in internalization (Fig 3E, 3F and 3K). Another phagocytic receptor has been studied in *Drosophila*, Croquemort (Crq) [18–20]. Indeed, we found that *crq* becomes up-regulated during mid- to late engulfment (Fig 4A–4C), suggesting it could be involved. To determine whether Crq could be acting as a third engulfment receptor in the follicle cells, we examined the ovaries of *crq*^*KO*^ mutants. The ovaries showed abnormalities in some egg chambers, where the follicle cells died prematurely, occasionally resulting in egg chambers completely devoid of follicle cells and undead germline (Fig 4D–4F) similar to what is seen when apoptotic genes are disrupted in the germline [42, 56, 57]. This severe phenotype did not occur in every egg chamber, and several healthy egg chambers were found with healthy follicle cells remaining. Also, when the nurse cells attempted to die, the chromatin did not fragment in the stereotypical fashion in phase 2 egg chambers. Often, the nurse cell nuclei formed a line, distinct from the disorganized chromatin normally seen in phase 1 and the tightly condensed chromatin seen in phase 3. This suggests that Crq may play a role in promoting nurse cell nuclear breakdown during nurse cell death.

**Fig 5:**
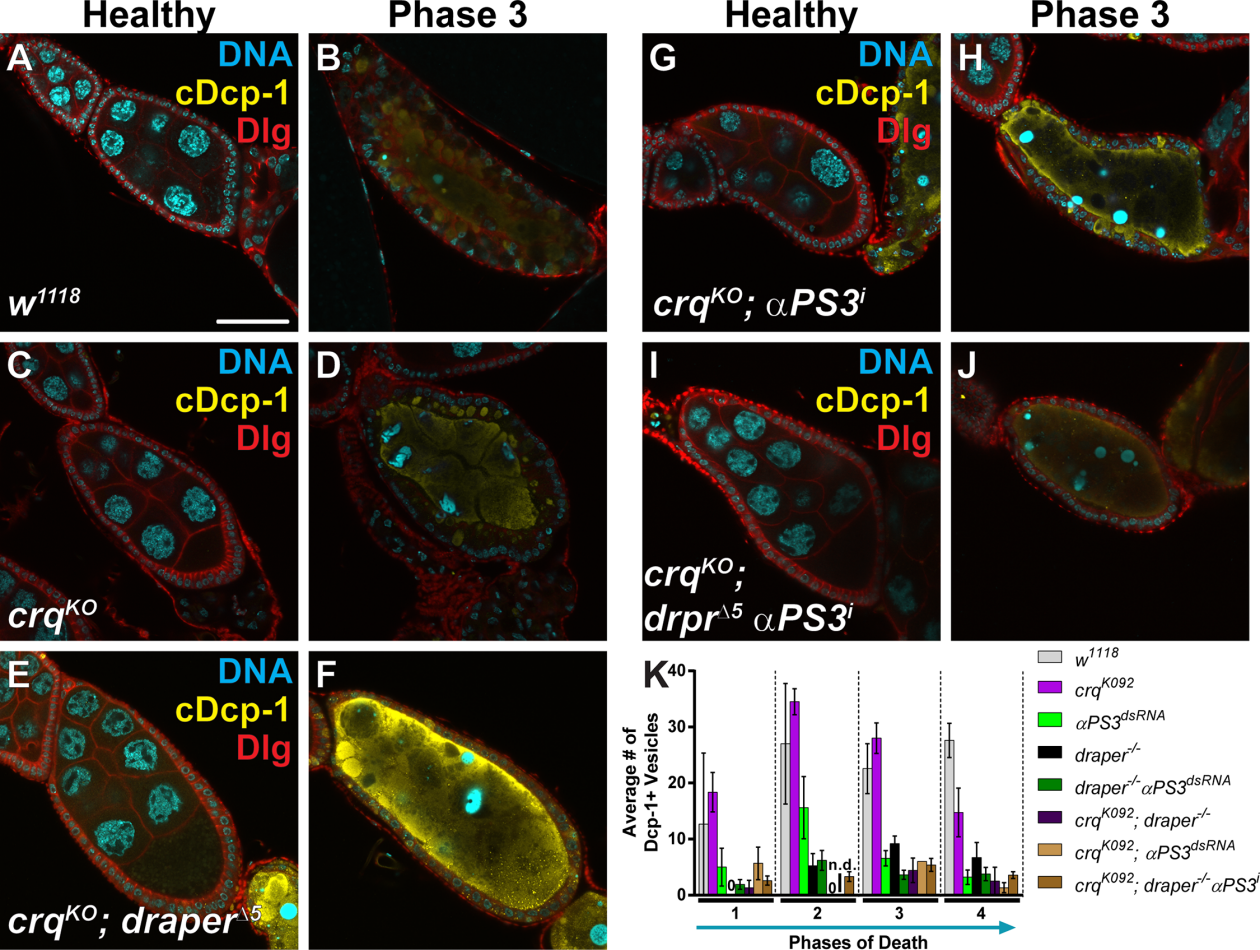
Croquemort is not required for engulfment by the follicle cells. (A-J) Mid-stage healthy and dying egg chambers from the indicated genotypes stained with DAPI (cyan) and antibodies against cleaved Dcp-1 (yellow) and Discs large (red). The Discs large channel was brightened in C-D and G-H to better visualize follicle cell enlargement. (A-B) Control (*w*^*1118*^) egg chambers show normal follicle cell enlargement and engulfment. (C-D) Loss of *crq* alone (*crq*^*KO*^) shows no defects in follicle cell enlargement or engulfment. (E-F) Loss of *crq* and *draper* (*crq*^*KO*^; *draper*^*Δ5*^) results in strong engulfment defects, similar to loss of *draper* alone. (G-H) Loss of *crq* and *αPS3* (*crq*^*KO*^*; GR1-GAL4/UAS-αPS3*^*dsRNA*^) results in strong engulfment defects, but not stronger than loss of *αPS3* alone. (I-J) Loss of *crq*, *draper*, and *αPS3* (*crq*^*KO*^*; draper*^*Δ5*^ *UAS-αPS3*^*dsRNA*^*/draper*^*Δ5*^, *GR1-GAL4*) results in strong engulfment defects, but not stronger than loss of *draper* and *αPS3*. (K) Average number of Dcp-1-positive vesicles engulfed per central slice for each phase of death. Scale bar is 50μm. All data are mean ± s.e.m. At least three egg chambers were quantified for each genotype and phase in K-M, except those noted here. For K, 19 egg chambers were quantified for *w*^*1118*^; 32 for *crq*^*KO*^; 44 for *αPS3*^*dsRNA*^; 46 for *draper*^*Δ5*^; 85 for *draper*^*Δ5*^ *αPS3*^*dsRNA*^; 20 for *crq*^*KO*^*; draper*^*Δ5*^; 7 for *crq*^*KO*^ *αPS3*^*dsRNA*^; 86 for *crq*^*KO*^*; draper*^*Δ5*^ *αPS3*^*dsRNA*^. Phase 2 for *crq*^*KO*^*; draper*^*Δ5*^ and *crq*^*KO*^*; αPS3*^*dsRNA*^ and phase 3 of *crq*^*KO*^*; αPS3*^*dsRNA*^ have less than 3 egg chambers quantified. The data for *αPS3*^*dsRNA*^, *draper*^*Δ5*^, and *draper*^*Δ5*^ *αPS3*^*dsRNA*^ were first shown in Fig 3. Two-tailed *t*-tests were performed: ***–*P*<0.005, **–*P*<0.01, *–*P*<0.05.

We stained *crq*^*KO*^ egg chambers with cleaved Dcp-1 and found that complete loss of *crq* resulted in no defects in vesicle uptake when compared to controls (Fig 5A–5D and 5K). As CD36 receptors can function together with other receptors, we performed experiments with double and triple mutants with αPS3 and Draper. Loss of either *crq* and *draper*, or *crq* and *αPS3*, resulted in no stronger defect than loss of either gene individually (Fig 5E–5H and 5K). Even the triple mutant with *draper* and *crq* null mutations combined with a knockdown of *αPS3* resulted in no additional defects (Fig 5I–5K). These results suggest that *crq* is not required in the follicle cells for engulfment, although it may be required for follicle cell survival or for promoting nurse cell chromatin changes during cell death. The residual engulfment that occurs in *draper*, *αPS3* double mutants may either indicate that another unknown engulfment receptor still acts in follicle cells, that there is an incomplete knockdown of *αPS3*, or that there is some engulfment that is not dependent on an engulfment receptor.

Because we did not find engulfment defects in *crq* mutants, we investigated other CD36 family receptors. We obtained knockdown or knockout lines of several CD36 receptors that are reported to be expressed in the ovary (FlyBase.org). We found that loss of *emp* or *santa-maria* did not have noticeable phenotypes. However, ovaries of *peste* mutants were grossly abnormal and resulted in significantly more “undead” egg chambers than seen in *crq* mutant ovaries, making it challenging to analyze engulfment. Another CD36 receptor previously reported to be required for engulfment, *debris buster*, is not expressed in the ovary, suggesting it is not required during oogenesis.

### Integrins and Draper function independently of each other during corpse processing

**Fig 6:**
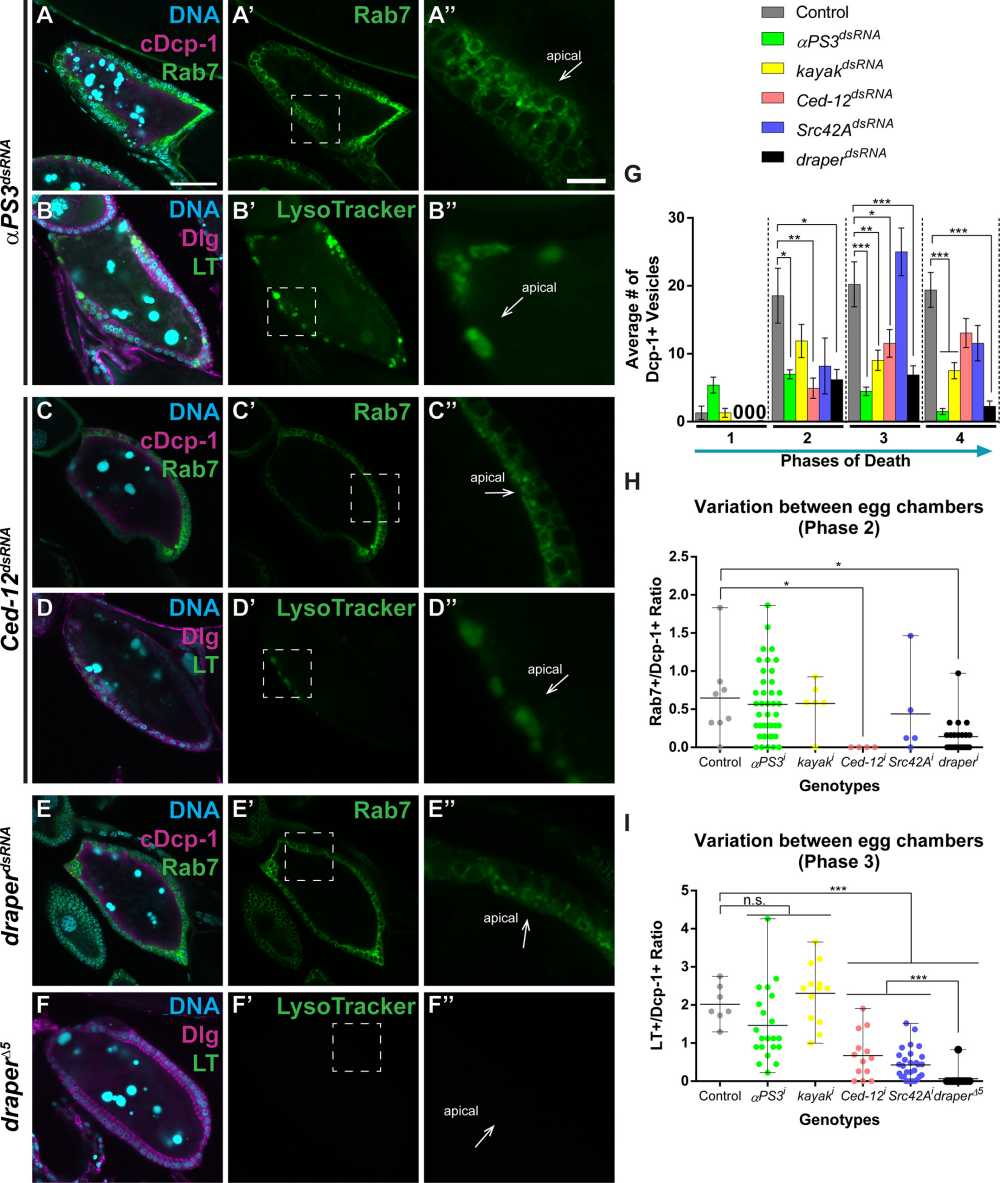
Engulfment mutants have distinct effects on corpse processing. (A-F) Dying egg chambers from the indicated genotypes stained with DAPI (cyan) and antibodies against cleaved Dcp-1 or Dlg (magenta). (A-A”) Loss of αPS3 (*UAS-GAL4/+; GR1-GAL4*, *UAS-Rab7GFP/UAS-αPS3*^*dsRNA*^) results in reduced vesicle uptake, but some of the vesicles become Rab7-positive. (B-B”) Loss of αPS3 (*UAS-GAL4/+; GR1-GAL4*, *UAS-Rab7GFP/UAS-αPS3*^*dsRNA*^) results in reduced vesicle uptake and normal acidification. (C-C”) Loss of *Ced-12* (*UAS-GAL4/+; GR1-GAL4*, *UAS-Rab7GFP/UAS-Ced-12*^*dsRNA*^) results in reduced vesicle uptake, reduced Rab7 association, and Rab7 aggregates. (D-D”) Loss of *Ced-12* (*UAS-GAL4/+; GR1-GAL4*, *UAS-Rab7GFP/UAS-Ced-12*^*dsRNA*^) results in reduced acidification. (E-E”) Loss of *draper* (*UAS-GAL4/UAS-draper*^*dsRNA*^*; GR1-GAL4*, *UAS-Rab7GFP/+*) results in reduced vesicle uptake, and very few vesicles are Rab7 positive. (F-F”) Loss of *draper (draper*^*Δ5*^*)* results in a severe delay in acidification, with little to no vesicles acidified at phase 3. (G) Average number of Dcp-1-positive vesicles engulfed per central slice. (H) Ratio of Rab7-positive to Dcp-1-positive vesicles in phase 2 egg chambers and (I) ratio of LysoTracker-positive vesicles to Dcp-1-positive vesicles in phase 3 egg chambers. Egg chamber scale bar is 50μm. Zoom scale bar is 10μm. All data are mean ± s.e.m. At least three egg chambers were quantified for each genotype, phase, and quantification method in G-I, except those noted here. For G, 47 egg chambers were quantified for the control (*UAS-GAL4/+; GR1-GAL4*, *UAS-Rab5GFP/UAS-luciferase*^*dsRNA*^ and *UAS-GAL4/+; GR1-GAL4*, *UAS-Rab7GFP/UAS-luciferase*^*dsRNA*^); 215 for *αPS3*^*dsRNA*^; 69 for *kayak*^*dsRNA*^; 82 for *Ced-12*^*dsRNA*^; 32 for *Src42A*^*dsRNA*^; and 73 for *draper*^*dsRNA*^. For H, 8 egg chambers were quantified for control (*UAS-GAL4/+; GR1-GAL4*, *UAS-Rab7GFP/UAS-luciferase*^*dsRNA*^); 41 for *αPS3*^*dsRNA*^; 6 for *kayak*^*dsRNA*^; 4 for *Ced-12*^*dsRNA*^; 5 for *Src42A*^*dsRNA*^; and 22 for *draper*^*dsRNA*^. For I, 7 egg chambers were quantified for control (the mixture from G); 21 for *αPS3*^*dsRNA*^; 13 for *kayak*^*dsRNA*^; 13 for *Ced-12*^*dsRNA*^; 29 for *Src42A*^*dsRNA*^; and 13 for *draper*^*Δ5*^. Phase 1 for *Src42A* has less than 3 egg chambers quantified. The data for control, *αPS3*^*dsRNA*^, *Ced-12*^*dsRNA*^, and *Src42A*^*dsRNA*^ were first shown in Fig 3. Two-tailed *t*-tests were performed: ***–*P*<0.005, **–*P*<0.01, *–*P*<0.05.

As Draper becomes internalized and may function in a partially parallel pathway to integrins, we next asked if these and other core engulfment proteins are required for phagosome maturation or acidification. Since Rab7 association was more discrete than Rab5 association (Fig 1), we used Rab7 to analyze potential defects in phagosome maturation in the engulfment machinery mutants (Fig 6). As all mutants had defects in internalization, we calculated the ratio of Rab7- to Dcp-1-positive vesicles to determine if fewer vesicles became Rab7-positive, indicating defects in phagosome maturation (Fig 6). This is based on the hypothesis that nascent phagosomes (Dcp-1+, Rab7-) mature to Dcp-1+, Rab7+ phagosomes. To analyze defects in acidification, we used LysoTracker, which marks acidified compartments. We also calculated the ratio of LysoTracker- to Dcp-1-positive vesicles to determine if fewer vesicles became acidified, indicating defects in acidification. Surprisingly, we found that engulfment defective mutants fell into three general categories in terms of defects in corpse processing. Loss of *αPS3* or *kayak* (JNK pathway transcription factor) resulted in defects in internalization, but no defects in the ratio of Rab7-positive vesicles (phagosome maturation) or the ratio of LysoTracker-positive vesicles (acidification) (Fig 6A–6B”,6G–6I). For example, loss of *αPS3* resulted in fewer Dcp-1-positive vesicles throughout engulfment (Fig 6G). However, the same ratio (approximately 70%) of those vesicles still became positive for Rab7, indicating that the vesicles taken up still progressed as in the control (Fig 6H). This holds true for acidification as well. The same ratio (approximately 180%) of those internalized vesicles became positive for LysoTracker, indicating that the vesicles taken up are still acidified as in the control (Fig 6I). Surprisingly, loss of *Ced-12* or *Src42A* resulted in not only defects in internalization, but also phagosome maturation (Fig 6C–6C”,6G and 6H). However, loss of *Ced-12* or *Src42A* resulted in milder defects in acidification compared to *draper* (Fig 6D–6D”,6I). Noticeably, the effect of the *Src42A* knockdown is weaker than that of *Ced-12*. This could be due to inefficient knockdowns or may speak to different requirements for these proteins. As seen in other systems, loss of *draper* resulted in defects in internalization, phagosome maturation, and acidification (Fig 6E–6F, 6G–6I). Indeed, LysoTracker labeling was almost completely abolished in *draper*^*Δ5*^ egg chambers (Fig 6F and 6I). Together, this suggests that some engulfment genes, such as *draper*, may be required for multiple steps during corpse processing while others, such as Ced-12, may be required for the efficiency of certain steps during corpse processing.

**Fig 7:**
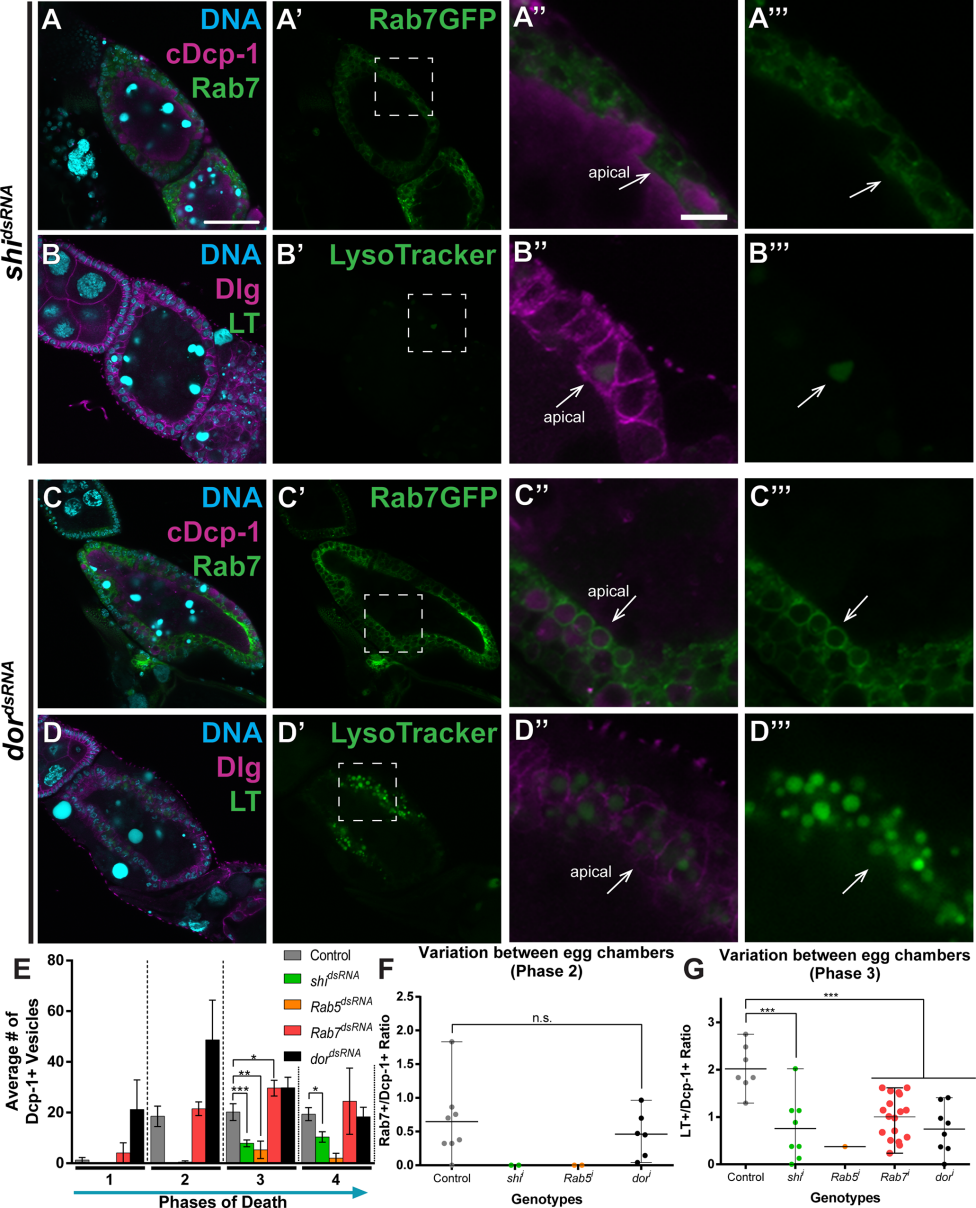
Loss of the canonical corpse processing genes, *shi* and *dor*, result in opposite defects. (A-D) Dying egg chambers from the indicated genotypes stained with DAPI (cyan) and antibodies against cleaved Dcp-1 or Dlg (magenta). (A-A”) Loss of *shi* (*UAS-GAL4/tub-GAL80*^*ts*^*; GR1-GAL4*, *UAS-Rab7GFP/UAS-shi*^*dsRNA*^) results in reduced vesicle uptake, and the few vesicles that are engulfed are not Rab7-positive. (B-B”) Loss of *shi* (*UAS-GAL4/tub-GAL80*^*ts*^*; GR1-GAL4*, *UAS-Rab7GFP/UAS-shi*^*dsRNA*^) results in little to no LysoTracker-positive vesicles. (C-C”) Loss of *dor* (*UAS-GAL4/+; GR1-GAL4*, *UAS-Rab7GFP/UAS-dor*^*dsRNA*^) results in elevated numbers of vesicles, and many are Rab7-positive. (D-D”) Loss of *dor* (*UAS-GAL4/+; GR1-GAL4*, *UAS-Rab7GFP/UAS-dor*^*dsRNA*^) results in elevated numbers of LysoTracker-positive vesicles. (E) Average number of Dcp-1-positive vesicles engulfed per central slice. (F) Ratio of Rab7-positive to Dcp-1-positive vesicles in phase 2 egg chambers and (G) ratio of LysoTracker-positive vesicles to Dcp-1-positive vesicles in phase 3 egg chambers. Egg chamber scale bar is 50μm. Zoom scale bar is 10μm. At least three egg chambers were quantified for each genotype, phase, and quantification method in E-G, except those noted here. For E, 47 egg chambers were quantified for the control (*UAS-GAL4/+; GR1-GAL4*, *UAS-Rab5GFP/UAS-luciferase*^*dsRNA*^ and *UAS-GAL4/+; GR1-GAL4*, *UAS-Rab7GFP/UAS-luciferase*^*dsRNA*^); 33 for *shi*^*dsRNA*^; 15 for *Rab5*^*dsRNA*^; 41 for *Rab7*^*dsRNA*^; and 48 for *dor*^*dsRNA*^. For F, 8 egg chambers were quantified for control (*UAS-GAL4/+; GR1-GAL4*, *UAS-Rab7GFP/UAS-luciferase*^*dsRNA*^); 2 for *shi*^*dsRNA*^; 2 for *Rab5*^*dsRNA*^; and 6 for *dor*^*dsRNA*^. For G, 7 egg chambers were quantified for control (the mixture from E); 8 for *shi*^*dsRNA*^; 1 for *Rab5*^*dsRNA*^; 18 for *Rab7*^*dsRNA*^; and 8 for *dor*^*dsRNA*^. Phase 2 for *shi*^*dsRNA*^ and *Rab5*^*dsRNA*^ and phase 4 for *Rab5*^*dsRNA*^ have less than 3 egg chambers quantified. Phase 3 for *Rab5*^*dsRNA*^, LT only, has less than 3 egg chambers quantified. The data for control were first shown in Fig 3. Two-tailed *t*-tests were performed: ***–*P*<0.005, **–*P*<0.01, *–*P*<0.05.

We also examined loss of known corpse processing genes *shibire* (*dynamin*–known to affect internalization), *Rab5* (early endosomes), *Rab7* (late endosomes), and *deep orange* (encoding VPS18, a lysosome biogenesis and fusion gene [58]). Loss of *shibire* and *Rab5* resulted in a defect very similar to loss of *draper*. Egg chambers expressing *shibire*^*dsRNA*^ or *Rab5*^*dsRNA*^ took up little to no vesicles, and those that were engulfed were not processed (Rab7-positive) or acidified (Fig 7A–7B”‘,7E–7G). Loss of *Rab7* (shown to be an effective RNAi knockdown in [59]) had no defects in internalization, but had a reduction in acidification (Fig 7E and 7G). Interestingly, loss of *dor* resulted in an elevated numbers of internalized vesicles, which were processed (became Rab7-positive) in the same proportions as controls. These vesicles also became acidified, but were often extremely small, resulting in a high number of small acidified vesicles within the follicle cells (Fig 7C–7G). However, the ratio of LysoTracker- to Dcp-1-positive vesicles was lower than that of control (Fig 7G). This suggests that loss of *dor* may result in a decrease in acidification, either resulting from defects in lysosome biogenesis or fusion to late endosomes.

## Discussion

Degradation of the cell corpse is crucial during engulfment. Here, we show that the epithelial follicle cells utilize the canonical corpse processing pathway to process Dcp-1-positive engulfed vesicles. Moreover, we show that engulfment receptors have distinct expression patterns during engulfment; Draper is present on the nascent, PI(3)P-positive phagosomes, whereas integrins are not. Using double mutant analysis, our data suggest that integrins may act through Src42A and Ced-12 in a pathway partially parallel to Draper during later phases of engulfment. However, our results suggest that Ced-12 is not initially activated by integrins, but rather may be activated by Draper. These conclusions about Src42A and Ced-12 could also be affected by inefficient knockdowns in the *GR1-GAL4*, *UAS-αPS3-dsRNA* recombinant or the double RNAi knockdowns.

Double *αPS3 draper* mutants had a very strong inhibition of engulfment, but it was not completely blocked. Surprisingly, the addition of the *crq* mutation to the *draper* and *αPS3* mutants did not result in a further block in engulfment. These results suggest that Draper and integrins are the main phagocytic receptors in the follicle cells. These results also suggest that another, yet unknown, engulfment receptor, may be required for engulfment.

Since loss of Ced-12 leads to defects in phagosome maturation but loss of integrins does not, this suggests that the role for Ced-12 in corpse processing is independent of integrins. Consistent with other systems, we found that *draper* mutants have defects in internalization, phagosome maturation, and acidification [37, 38]. In contrast, knockdowns of the JNK pathway transcription factor, Kayak, only have defects in internalization. Our data suggest that there may be three categories of defects: 1) internalization only (*αPS3*, *kayak*); 2) internalization and phagosome maturation (*Ced-12*, *Src42A*); and 3) internalization, phagosome maturation, and acidification (*draper*). However, given that we quantified the acidification defects in a *draper* null mutant and used RNAi to knockdown *Ced-12* and *Src42A*, it is possible that one or both of these genes are also required for acidification and this phenotype would only be apparent with a null mutant. The defect we see in *kayak* RNAi egg chambers suggests that the role of the JNK pathway is in signal amplification, and not in corpse processing during engulfment. This is consistent with our findings that loss of *kayak* and basket (JNK [45]) only result in significant defects by phase 3 of engulfment. To our knowledge, we provide the first systematic study of corpse processing in an epithelial layer and an investigation into the cross-talk between several core engulfment machinery components and corpse processing.

**Fig 8:**
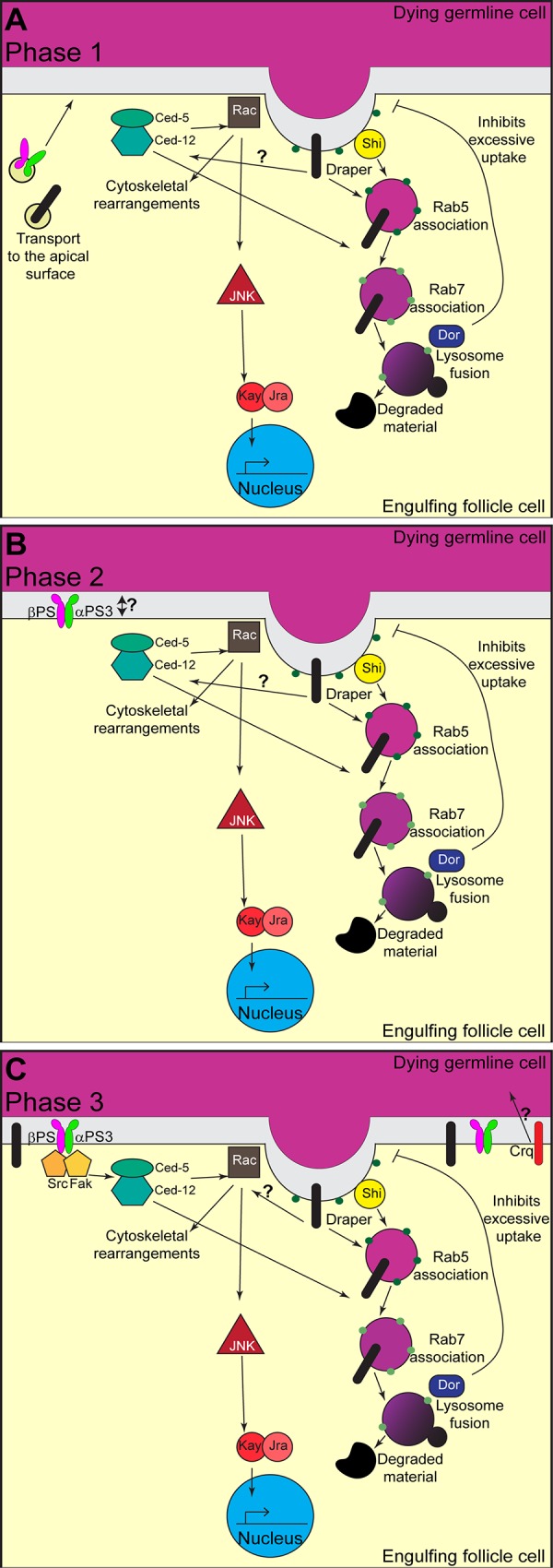
Model of engulfment throughout the initial phases of engulfment. A diagram depicting our model for the molecular changes within an engulfing epithelial follicle cell. (A) In phase 1 egg chambers, Draper is already present on the apical surface of the follicle cells and initiates corpse processing and activation of Rac1 by activating Ced-12. Ced-12 is required for efficient corpse processing. Integrins and Draper are also being trafficked to the apical surface and Dor activity is required for inhibiting excessive uptake. (B) In phase 2 egg chambers, integrins are potentially required for adhesion to the dying germline. (C) In phase 3 egg chambers, both integrins and Draper are fully active and working in concert to promote engulfment. The JNK pathway is active and serves as an amplification signal, increasing engulfment genes such as Draper.

We propose the following model (Fig 8) for cross-talk between engulfment and corpse processing machinery in the follicular epithelium: in early dying phase 1 egg chambers, Draper is present on the phagocytic cup and nascent phagosomes and is required for internalization, phagosome maturation, acidification, as well as signaling to other engulfment machinery. As Draper is present on healthy follicle cells, it is responsible for the majority of the internalization in phase 1. Also in phase 1 egg chambers, we propose that Ced-12 is activated by Draper and is required for phagosome maturation. Consistent with this, integrin mutants do not show engulfment defects in phase 1 egg chambers [14]. By phase 2, integrins are present on the apical surface and may play a role in anchoring the engulfing cell to the dying cell, as shown in RPE cells [60, 61]. By phase 3, Draper and integrins are both active and appear to work in concert to regulate internalization and engulfment. During this phase of engulfment, JNK signaling is activated and has amplified the engulfment signal, at least in part by producing more Draper for internalization. Consistent with other systems, integrins may work through Src42A and Ced-12 during mid- to late engulfment.

We found it striking that the core engulfment machinery has distinct temporal regulation and different functions within the engulfing cell. One possibility is that different categories of proteins (receptors, kinases, adaptor proteins, etc.) inherently play different roles within engulfment. However, two engulfment receptors (Draper and integrins) have very different functions in terms of corpse processing while Src42A and Ced-12 (a tyrosine kinase and adaptor protein, respectively) are both required for efficient phagosome maturation. Another possibility is that the proteins required for corpse processing are localized to phagosomes. While loss of *Ced-12* and *Src42A* resulted in less efficient phagosome maturation, we do not know if these proteins localize to maturing phagosomes. This will only become clear with further investigation.

The regulation of these proteins is another point of interest. As mentioned above, we do not know if Ced-12 and Src42A directly localize to phagosomes or not. Phosphatidylinositol (PI) can be differentially phosphorylated, which may serve as a regulator for interaction with some, or all, of these proteins. As we have seen, Draper is present on several phagosomes that are PI(3)P-positive, suggesting that PI(3)P may be required to maintain Draper on nascent phagosomes. However, Draper is also present on some PI(3)P-negative phagosomes, suggesting either that Draper is present on less mature phagosomes or Draper-positive, Rab7- and PI(3)P-negative vesicles are recycling endosomes, allowing for Draper to be recycled back to the apical surface. In *C*. *elegans*, the phagocytic cup is marked by PI(4,5)P_2_ while the sealed and nascent phagosomes are marked by PI(3)P [31]. Sorting nexins have been shown to associate with certain phosphorylated forms of PI, but not others, and their presence on phagosomes affects their maturation [35]. Association of K-Ras with endosomes has been shown to be dependent on endosomal acidic phospholipids, specifically phosphatidylserine [62]. Lipid composition may be a more general method of recruiting proteins to the appropriate vesicle membrane for efficient phagosome maturation.

Engulfment by epithelial cells is crucial for the health and maintenance of several organs throughout an organism; however, very little is known about how epithelial cells degrade the engulfed material. In this study, we show that most, but not all, of the engulfment machinery is required for efficient corpse processing. Draper is present on the phagocytic cup and PI(3)P-positive vesicles, suggesting a possible method of recruitment of proteins, such as Src42A and Ced-12, to the proper phagosome membrane for efficient fusion with endosomes. This work suggests that the core engulfment machinery may play several roles within a cell, including internalization, corpse processing, acidification, and downstream signaling. We have established the *Drosophila* ovary as a powerful model for investigating the cross-talk between engulfment and corpse processing machinery in an in vivo system. There are striking similarities between the *Drosophila* epithelial follicle cells and mammalian retinal pigment epithelial cells [14], suggesting that the information gained here may be useful for diseases such as retinitis pigmentosa and age-related macular degeneration.

## Supporting Information

S1 Fig αPS3 is knocked down in all lines used for analysis. Dying mid-stage egg chambers from the indicated genotypes stained with DAPI (cyan) and an antibody against αPS3 (green). (A-A’) Egg chambers from wild-type (*w*^*1118*^) flies show enrichment of αPS3 (white) on the apical surface of the follicle cells. (B-B’) Knockdown of αPS3 (*GR1-GAL4/UAS-αPS3*^*dsRNA*^) in the follicle cells show no enrichment of αPS3. (C-C’) A *GR1-GAL4*, *UAS-αPS3*^*dsRNA*^ recombinant (*UAS-GAL4/+; GR1-GAL4*, *UAS-αPS3*^*dsRNA*^*/UAS-luciferase*^*dsRNA*^) also shows no enrichment. Scale bar is 50μm.
(TIF)

## References

[1] Patel V.A., Lee D.J., Feng L., Antoni A., Lieberthal W., Schwartz J.H., et al Recognition of apoptotic cells by epithelial cells: conserved versus tissue-specific signaling responses. J Biol Chem. 2010; 285: p. 1829–40. 10.1074/jbc.M109.018440 19910463 PMC2804341

[2] Nandrot E.F. and Finnemann S.C. Lack of alphavbeta5 integrin receptor or its ligand MFG-E8: distinct effects on retinal function. Ophthalmic Res. 2008; 40: p. 120–3. 10.1159/000119861 18421224 PMC3237195

[3] Juncadella I.J., Kadl A., Sharma A.K., Shim Y.M., Hochreiter-Hufford A., Borish L., et al Apoptotic cell clearance by bronchial epithelial cells critically influences airway inflammation. Nature. 2013; 493: p. 547–51. 10.1038/nature11714 23235830 PMC3662023

[4] Penberthy K.K., Juncadella I.J., and Ravichandran K.S. Apoptosis and engulfment by bronchial epithelial cells. Implications for allergic airway inflammation. Ann Am Thorac Soc. 2014; 11 Suppl 5: p. S259–62. 10.1513/AnnalsATS.201405-200AW 25525729 PMC4298971

[5] Nandrot E.F. Animal Models, in "*The Quest to Decipher RPE Phagocytosis*". Adv Exp Med Biol. 2014; 801: p. 77–83. 10.1007/978-1-4614-3209-8_10 24664683

[6] Hsieh H.-H., Hsu T.-Y., Jiang H.-S., and Wu Y.-C. Integrin α PAT-2/CDC-42 Signaling Is Required for Muscle-Mediated Clearance of Apoptotic Cells in *Caenorhabditis elegans*. PLoS Genet. 2012; 8: p. 10.1371/journal.pgen.1002663 PMC3355063 22615577

[7] Neukomm L.J., Zeng S., Frei A.P., Huegli P.A., and Hengartner M.O. Small GTPase CDC-42 promotes apoptotic cell corpse clearance in response to PAT-2 and CED-1 in C. elegans. Cell Death Differ. 2014; 10.1038/cdd.2014.23 PMC4013519 24632947

[8] Kinchen J.M., Cabello J., Klingele D., Wong K., Feichtinger R., Schnabel H., et al Two pathways coverege at CED-10 to mediate actin rearrangement and corpse removal in *C*. *elegans*. Nature. 2005; 434: p. 93–9. 15744306 10.1038/nature03263

[9] Ziegenfuss J.S., Doherty J., and Freeman M.R. Distinct molecular pathways mediate glial activation and engulfment of axonal debris after axotomy. Nat Neurosci. 2012; 15: p. 979–87. 10.1038/nn.3135 22706267 PMC4976689

[10] Lu T.-Y., Doherty J., and Freeman M.R. DRK/DOS/SOS converge with Crk/Mbc/dCed-12 to activate Rac1 during glial engulfment of axonal debris. Proc Natl Acad Sci U S A. 2014; 111: p. 12544–9. 10.1073/pnas.1403450111 25099352 PMC4151738

[11] Shiratsuchi A., Mori T., Sakurai K., Nagaosa K., Sekimizu K., Lee B.L., et al Independent Recognition of *Staphylococcus aureus* by Two Receptors for Phagocytosis in *Drosophila*. J Biol Chem. 2012; 287: p. 21663–72. 10.1074/jbc.M111.333807 22547074 PMC3381130

[12] Nonaka S., Nagaosa K., Mori T., Shiratsuchi A., and Nakanishi Y. Integrin αPS3/βν-mediated Phagocytosis of Apoptotic Cells and Bacteria in *Drosophila*. J Biol Chem. 2013; 288: p. 10374–80. 10.1074/jbc.M113.451427 23426364 PMC3624420

[13] Nagaosa K., Okada R., Nonaka S., Takeuchi K., Fujita Y., Miyasaka T., et al Integrin βν-mediated Phagocytosis of Apoptotic Cells in *Drosophila* Embryos. J Biol Chem. 2011; 286: p. 25770–7. 10.1074/jbc.M110.204503 21592968 PMC3138285

[14] Meehan T.L., Kleinsorge S.E., Timmons A.K., Taylor J.D., and McCall K. Polarization of the epithelial layer and apical localization of integrins are required for engulfment of apoptotic cells. Dis Model Mech. 2015; 8: p1603–14. 10.1242/dmm.021998 PMC4728319 26398951

[15] Van Goethem E., Silva E.A., Xiao H., and Franc N.C. The Drosophila TRPP cation channel, PKD2 and Dmel/Ced-12 act in genetically distinct pathways during apoptotic cell clearance. PLoS One. 2012; 7: p. e31488 10.1371/journal.pone.0031488 22347485 PMC3275576

[16] Das S., Owen K.A., Ly K.T., Park D., Black S.G., Wilson J.M., et al Brain angiogenesis inhibitor (BAI1) is a pattern recognition receptor that mediates macrophage binding and engulfment of Gram-negative bacteria. Proc Natl Acad Sci U S A. 2011; 108: p. 2136–41. 10.1073/pnas.1014775108 21245295 PMC3033312

[17] Fond A.M., Lee C.S., Schulman I.G., Kiss R.S., and Ravichandran K.S. Apoptotic cells trigger a membrane-initiated pathway to increase ABCA1. J Clin Invest. 2015; 125: p. 2748–58. 10.1172/JCI80300 26075824 PMC4563683

[18] Franc N.C., Dimarcq J.L., Laqueux M., Koffmann J., and Ezekowitz R.A. Croquemort, a novel *Drosophila* hemocyte/macrophage receptor that recognizes apoptotic cells. Immun. 1996; 4: p. 431–443. 10.1016/s1074-7613(00)80410-0 8630729

[19] Franc N.C. Requirement for Croquemort in Phagocytosis of Apoptotic Cells in Drosophila. Science. 1999; 284: p. 1991–1994. 10373118 10.1126/science.284.5422.1991

[20] Han C., Song Y., Xiao H., Wang D., Franc N.C., Jan L.Y., et al Epidermal cells are the primary phagocytes in the fragmentation and clearance of degenerating dendrites in *Drosophila*. Neuron. 2014; 81: p. 544–60. 10.1016/j.neuron.2013.11.021 24412417 PMC3995171

[21] Philips J.A., Rubin E., and Perrimon N. *Drosophila* RNAi screen reveals CD36 family member required for mycobacterial infection. Science. 2005; 309: p. 1251–3. 16020694 10.1126/science.1116006

[22] Yu X., Odera S., Chuang C.H., Lu N., and Zhou Z. C. elegans Dynamin mediates the signaling of phagocytic receptor CED-1 for the engulfment and degradation of apoptotic cells. Dev Cell. 2006; 10: p. 743–57. 16740477 10.1016/j.devcel.2006.04.007

[23] Kinchen J.M., Doukoumetzidis K., Almendinger J., Stergiou L., Tosello-Trampont A., Sifri C.D., et al A pathway for phagosome maturation during engulfment of apoptotic cells. Nat Cell Biol. 2008; 10: p. 556–66. 10.1038/ncb1718 18425118 PMC2851549

[24] Vieira O.V., Bucci C., Harrison R.E., Trimble W.S., Lanzetti L., Gruenberg J., et al Modulation of Rab5 and Rab7 recruitment to phagosomes by phosphatidylinositol 3-kinase. Mol Biol Cell. 2003; 23: p. 2501–14. 10.1128/MCB.23.7.2501-2514.2003 PMC150733 12640132

[25] Sasaki A., Nakae I., Nagasawa M., Hashimoto K., Abe F., Saito K., et al Arl8/ARL-8 functions in apoptotic cell removal by mediating phagolysosome formation in Caenorhabditis elegans. Mol Biol Cell. 2013; 24: p. 1584–92. 10.1091/mbc.E12-08-0628 23485564 PMC3655818

[26] Nakae I., Fujino T., Kobayashi T., Sasaki A., Kikko Y., Fukuyama m., et al The Arf-like GTPase Arl8 Mediates Delivery of Endocytosed Macromolecules to Lysosomes in *Caenorhabditis elegans*. Mol Biol Cell. 2010; 21: p. 2434–42. 10.1091/mbc.E09-12-1010 20484575 PMC2903672

[27] Lukacs G.L., Rotstein O.D., and Grinstein S. Phagosomal acidification is mediated by a vacuolar-type H+-ATPase in murine macrophages. J Biol Chem. 1990; 265: p. 21099–107. 2147429

[28] Huynh K.K., Eskelinen E.-L., Scott C.C., Malevanets A., Saftig P., and Grinstein S. LAMP proteins are required for fusion of lysosomes with phagosomes. EMBO J. 2007; 26: p. 313–24. 17245426 10.1038/sj.emboj.7601511 PMC1783450

[29] Lu N., Shen Q., Mahoney T.R., Neukomm L.J., Wang Y., and Zhou Z. Two PI 3-kinases and one PI 3-phosphatase together establish the cyclic waves of phagosomal PtdIns(3)P critical for the degradation of apoptotic cells. PLoS Biol. 2012; 10: p. e1001245 10.1371/journal.pbio.1001245 22272187 PMC3260314

[30] Vieira O.V., Botelho R.J., Rameh L., Brachmann S.M., Matsuo T., Davidson H.W., et al Distinct roles of class I and class III phosphatidylinositol 3-kinases in phagosome formation and maturation. J Cell Biol. 2001; 155: p. 19–26. 11581283 10.1083/jcb.200107069 PMC2150784

[31] Cheng S., Wang K., Zou W., Miao R., Huang Y., Wang H., et al PtdIns(4,5)P2 and PtdIns3P coordinate to regulate phagosomal sealing for apoptotic cell clearance. J Cell Biol. 2015; 210: p. 485–502. 10.1083/jcb.201501038 26240185 PMC4523610

[32] Shandala T., Lim C., Sorvina A., and Brooks D.A. A *Drosophila* model to image phagosome maturation. Cells. 2013; 2: p. 188–201. 10.3390/cells2020188 24709696 PMC3972680

[33] Meehan T.L., Yalonetskaya A., Joudi T.F., and McCall K. Detection of cell death and phagocytosis in the *Drosophila* ovary. Methods Mol Biol. 2015; 1328: p. 191–206. 10.1007/978-1-4939-2851-4_14 26324439

[34] Yu X., Lu N., and Zhou Z. Phagocytic receptor CED-1 initiates a signaling pathway for degrading engulfed apoptotic cells. PLoS One. 2008; 6: p. e61. 10.1371/journal.pbio.0060061 PMC2267821 18351800

[35] Lu N., Shen Q., Mahoney T.R., Liu X., and Zhou Z. Three sorting nexins drive the degradation of apoptotic cells in response to PtdIns(3)P signaling. Mol Biol Cell. 2011; 22: p. 354–74. 10.1091/mbc.E10-09-0756 21148288 PMC3031466

[36] Chen D., Jian Y., Liu X., Zhang Y., Liang J., Qi X., et al Clathrin and AP2 are required for phagocytic receptor-mediated apoptotic cell clearance in Caenorhabditis elegans. PLoS Genet. 2013; 9: p. e1003517 10.1371/journal.pgen.1003517 23696751 PMC3656144

[37] Evans I.R., Rodrigues F.S., Armitage E.L., and Wood W. Draper/CEd-1 mediates an ancient damage response to control inflammatory blood cell migration in vivo. Curr Biol. 2015; 25: p. 1606–12. 10.1016/j.cub.2015.04.037 26028435 PMC4503800

[38] Kurant E., Axelrod S., Leaman D., and Gaul U. Six-microns-under acts upstream of Draper in the glial phagocytosis of apoptotic neurons. Cell. 2008; 133: p. 498–509. 10.1016/j.cell.2008.02.052 18455990 PMC2730188

[39] Giorgi F. and Deri P. Cell death in ovarian chambers of *Drosophila melanogaster*. J. Embryol. Exp. Morph. 1976; 35: p. 521–533. 820828

[40] Nezis I.P., Stravopodis D.J., Papassideri I., Robert-Nicoud M., and Margaritis L.H. Stage-specific apoptotic patterns during Drosophila oogenesis. Eur J Cell Biol. 2000; 79: p. 610–20. 11043402 10.1078/0171-9335-00088

[41] Drummond-Barbosa D. and Spradling A.C. Stem cells and their progeny respond to nutritional changes during Drosophila oogenesis. Dev Biol. 2001; 231: p. 265–78. 11180967 10.1006/dbio.2000.0135

[42] Mazzalupo S. and Cooley L. Illuminating the role of caspases during Drosophila oogenesis. Cell Death Differ. 2006; 13: p. 1950–9. 16528381 10.1038/sj.cdd.4401892

[43] Hou Y.C., Chittaranjan S., Barbosa S.G., McCall K., and Gorski S.M. Effector caspase Dcp-1 and IAP protein Bruce regulate starvation-induced autophagy during Drosophila melanogaster oogenesis. J Cell Biol. 2008; 182: p. 1127–39. 10.1083/jcb.200712091 18794330 PMC2542474

[44] Tanner E.A., Blute T.A., Brachmann C.B., and McCall K. Bcl-2 proteins and autophagy regulate mitochondrial dynamics during programmed cell death in the *Drosophila* ovary. Development. 2011; 138: p. 327–338. 10.1242/dev.057943 21177345 PMC3005606

[45] Etchegaray J.I., Timmons A.K., Klein A.P., Pritchett T.L., Welch E., Meehan T.L., et al Draper acts through the JNK pathway to control synchronous engulfment of dying germline cells by follicular epithelial cells. Development. 2012; 139: p. 4029–39. 10.1242/dev.082776 22992958 PMC3472587

[46] Ni J.Q., Markstein M., Binari R., Pfeiffer B., Liu L.P., Villalta C., et al Vector and parameters for targeted transgenic RNA inteference in *Drosophila melanogaster*. Nat Methods. 2008; 5: p. 49–51. 18084299 10.1038/nmeth1146 PMC2290002

[47] Goentoro L.A., Yakoby N., Goodhouse J., Schupbach T., and Shvartsman S.Y. Quantitative analysis of the GAL4/UAS system in Drosophila oogenesis. Genesis. 2006; 44: p. 66–74. 16425298 10.1002/gene.20184

[48] Freeman M.R., Delrow J., Kim J., Johnson E., and Doe C.Q. Unwrapping glial biology: Gcm target genes regulating glial development, diversification, and function. Neuron. 2003; 38: p. 567–80. 12765609 10.1016/s0896-6273(03)00289-7

[49] Velichkova M., Juan J., Kadandale P., Jean S., Ribeiro I., Raman V., et al *Drosophila* Mtm and class II PI3K coregulate a PI(3)P pool with cortical and endolysosomal functions. J Cell Biol. 2010; 190: p. 407–25. 10.1083/jcb.200911020 20696708 PMC2922644

[50] Sarkissian T., Timmons A., Arya R., Abdelwahid E., and White K. Detecting apoptosis in Drosophila tissues and cells. Methods. 2014; 68: p. 89–96. 10.1016/j.ymeth.2014.02.033 24613678 PMC4048790

[51] Wucherpfennig T., Wilsch-Brauninger M., and Gonzalez-Gaitan M. Role of Drosophila Rab5 during endosomal trafficking at the synapse and evoked neurotransmitter release. J Cell Biol. 2003; 161: p. 609–24. 12743108 10.1083/jcb.200211087 PMC2172938

[52] Entchev E.V., Schwabedissen A., and Gonzalez-Gaitan M. Gradient formation of the TGF-β homolog Dpp. Cell. 2000; 103: p. 981–992. 11136982 10.1016/s0092-8674(00)00200-2

[53] Zhou Z., Hartwieg E., and Horvitz H.R. CED-1 is a transmembrane receptor that mediates cell corpse engulfment in *C*. *elegans*. Cell. 2001; 104: p. 43–56. 11163239 10.1016/s0092-8674(01)00190-8

[54] Manaka J., Kuraishi T., Shiratsuchi A., Nakai Y., Higashida H., Henson P., et al Draper-mediated and phosphatidylserine-independent phagocytosis of apoptotic cells by *Drosophila* hemocytes/macrophages. J Biol Chem. 2004; 279: p. 48466–76. 15342648 10.1074/jbc.M408597200

[55] Ziegenfuss J.S., Biswas R., Avery M.A., Hong K., Sheehan A.E., Yeung Y.-G., et al Draper-dependent glial phagocytic activity is mediated by Src and Syk family kinase signalling. Nature. 2008; 453: p. 935–9. 10.1038/nature06901 18432193 PMC2493287

[56] Peterson J.S., Barkett M., and McCall K. Stage-specific regulation of caspase activity in drosophila oogenesis. Developmental Biology. 2003; 260: p. 113–123. 12885559 10.1016/s0012-1606(03)00240-9

[57] Baum J.S., Arama E., Steller H., and McCall K. The *Drosophila* caspases Strica and Dronc function redundantly in programmed cell death during oogenesis. Cell Death Differ. 2007; 14: p. 1508–1517. 17464325 10.1038/sj.cdd.4402155

[58] Xiao H., Chen D., Fang Z., Xu J., Sun X., Song S., et al Lysosome biogenesis mediated by *vps-18* affects apoptotic cell degradation in *Caenorhabditis elegans*. Mol Biol Cell. 2008; 20: p. 21–32. 10.1091/mbc.E08-04-0441 18923146 PMC2613118

[59] Timmons, A.K., *Phagocytosis genes and the JNK signaling pathway promote developmental programmed cell death and are essential for the engulfment of the dying germline in the Drosophila ovary*. 2015.

[60] Finnemann S.C., Bonilha V.L., Marmorstein A.D., and Rodriguez-Boulan E. Phagocytosis of rod outer segments by retinal pigment epithelial cells requires ανβ5 integrin for binding but not for internalization. Proc Natl Acad Sci U S A. 1997; 94: p. 12932–37. 9371778 10.1073/pnas.94.24.12932 PMC24241

[61] Nandrot E.F., Kim Y., Brodie S.E., Huang X., Sheppard D., and Finnemann S.C. Loss of synchronized retinal phagocytosis and age-related blindness in mice lacking alphavbeta5 integrin. J Exp Med. 2004; 200: p. 1539–45. 15596525 10.1084/jem.20041447 PMC2211990

[62] Gelabert-Baldrich M., Soriano-Castell D., Calvo M., Lu A., Vina-Vilaseca A., Rentero C., et al Dynamics of KRas on endosomes: involvement of acidic phospholipids in its association. The FASEB Journal. 2014; 28: p. 3023–37. 10.1096/fj.13-241158 24719356

