# Supplementary figures and images for: Components of the Engulfment Machinery Have Distinct Roles in Corpse Processing
Source: PLoS One. 2016 Jun 27;11(6):e0158217. doi: 10.1371/journal.pone.0158217 (PMC4922577; doi:10.1371/journal.pone.0158217)

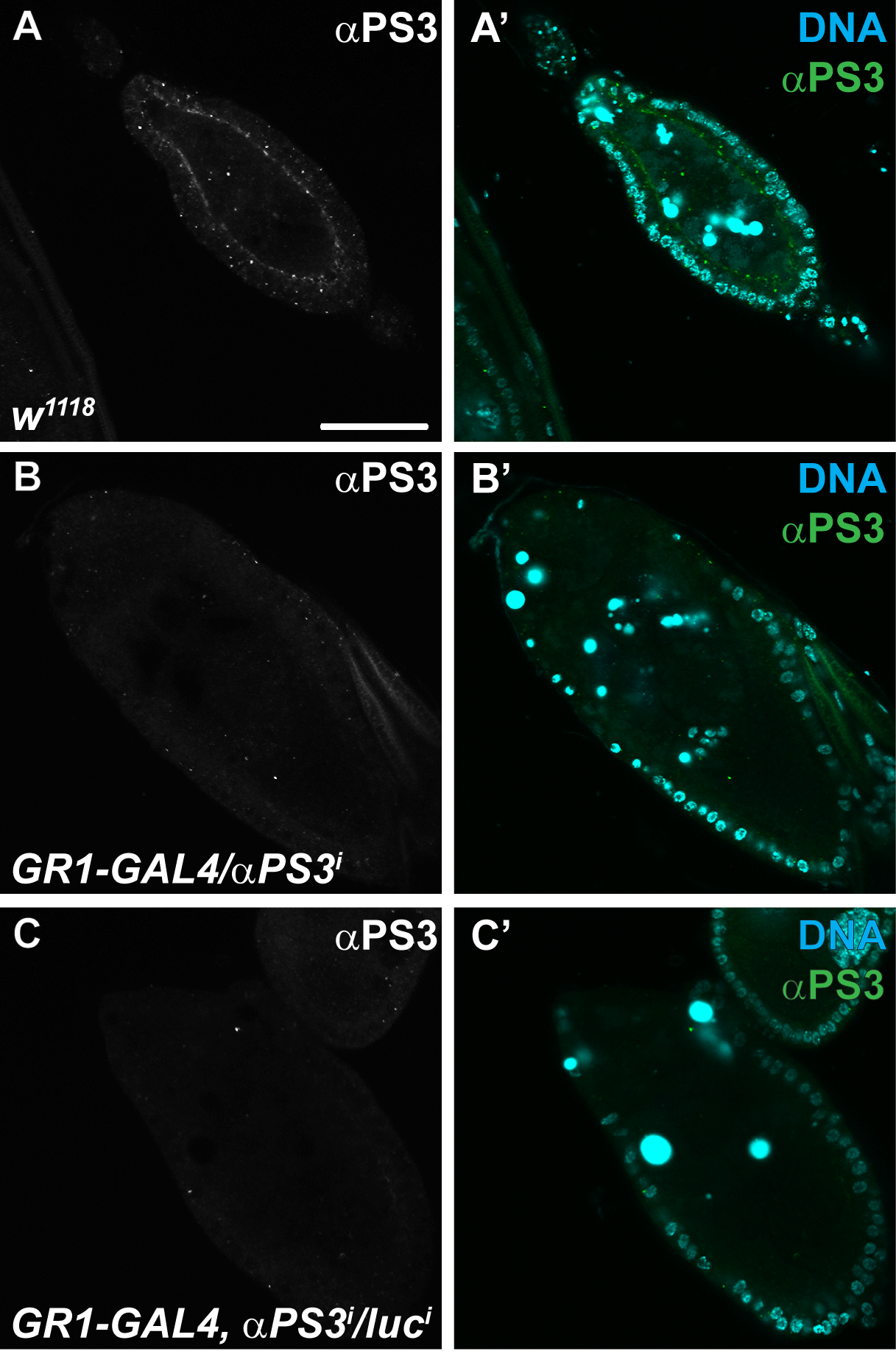

Supplement: S1 Fig — Dying mid-stage egg chambers from the indicated genotypes stained with DAPI (cyan) and an antibody against αPS3 (green). (A-A’) Egg chambers from wild-type (w1118) flies show enrichment of αPS3 (white) on the apical surface of the follicle cells. (B-B’) Knockdown of αPS3 (GR1-GAL4/UAS-αPS3dsRNA) in the follicle cells show no enrichment of αPS3. (C-C’) A GR1-GAL4, UAS-αPS3dsRNA recombinant (UAS-GAL4/+; GR1-GAL4, UAS-αPS3dsRNA/UAS-luciferasedsRNA) also shows no enrichment. Scale bar is 50μm. (TIF) [file pone.0158217.s001.tif]
